# Molecular, Biochemical, and Dietary Regulation Features of α-Amylase in a Carnivorous Crustacean, the Spiny Lobster *Panulirus argus*

**DOI:** 10.1371/journal.pone.0158919

**Published:** 2016-07-08

**Authors:** Leandro Rodríguez-Viera, Erick Perera, Juan Antonio Martos-Sitcha, Rolando Perdomo-Morales, Antonio Casuso, Vivian Montero-Alejo, Tsai García-Galano, Gonzalo Martínez-Rodríguez, Juan Miguel Mancera

**Affiliations:** 1 Center for Marine Research, University of Havana, Havana, Cuba; 2 Instituto de Ciencias Marinas de Andalucía, ICMAN-CSIC, Puerto Real, Cadiz, Spain; 3 Department of Biochemistry, Center for Pharmaceuticals Research and Development, Havana, Cuba; 4 Department of Biology, University of Cadiz, Puerto Real, Cadiz, Spain; Istituto Biologia e Biotecnologia Agraria IBBA, ITALY

## Abstract

Alpha-amylases are ubiquitously distributed throughout microbials, plants and animals. It is widely accepted that omnivorous crustaceans have higher α-amylase activity and number of isoforms than carnivorous, but contradictory results have been obtained in some species, and carnivorous crustaceans have been less studied. In addition, the physiological meaning of α-amylase polymorphism in crustaceans is not well understood. In this work we studied α-amylase in a carnivorous lobster at the gene, transcript, and protein levels. It was showed that α-amylase isoenzyme composition (i.e., phenotype) in lobster determines carbohydrate digestion efficiency. Most frequent α-amylase phenotype has the lowest digestion efficiency, suggesting this is a favoured trait. We revealed that gene and intron loss have occurred in lobster α-amylase, thus lobsters express a single 1830 bp cDNA encoding a highly conserved protein with 513 amino acids. This protein gives rise to two isoenzymes in some individuals by glycosylation but not by limited proteolysis. Only the glycosylated isoenzyme could be purified by chromatography, with biochemical features similar to other animal amylases. High carbohydrate content in diet down-regulates α-amylase gene expression in lobster. However, high α-amylase activity occurs in lobster gastric juice irrespective of diet and was proposed to function as an early sensor of the carbohydrate content of diet to regulate further gene expression. We concluded that gene/isoenzyme simplicity, post-translational modifications and low *K*m, coupled with a tight regulation of gene expression, have arose during evolution of α-amylase in the carnivorous lobster to control excessive carbohydrate digestion in the presence of an active α-amylase.

## Introduction

Different to other crustaceans that exhibit transitions in their feeding habits through ontogeny, spiny lobsters are carnivorous since first feeding. After hatching, spiny lobster larvae (phyllosoma) drift in ocean waters feeding on zooplankton preys such as salps, ctenophores, and medusae [1–3]. After metamorphosis and shelf settlement, postlarva, juveniles, and adult lobsters fed on a wide variety of benthic and infaunal species, such as gastropods, bivalves, chitons, anomurans, brachyurans, polychaete worms, and sea urchins, although they ingest occasional amounts of macroalgae and seaweed [4,5]. The chemical composition of this natural diet contains high protein, low lipid and moderate carbohydrate contents [6,7]. However, while the carnivorous behavior of spiny lobsters and the high protein content in their diet correlate well with the high proteinase activity they exhibit in the digestive tract [8, 9–11], the relationship between dietary carbohydrate and digestive carbohydrases in lobsters is less understood.

Alpha-amylase (α-1,4-alpha-D-glucan glucanohydrolase, EC 3.2.1.1) is responsible for the hydrolysis of α-1,4 glycoside bonds in starch and glycogen and it is the most important carbohydrases in lobsters [8,9,11]. Positive correlation of α-amylase activity and dietary carbohydrates is typical in very distant groups such as insects [12], mollusks [13], fish [14], dogs [15], and humans [16]. In general, high amylolytic activity in herbivorous and omnivorous is accepted to result from adaptation to low energy food and low assimilation efficiency or as an adaptation to large amounts of dietary starch [17]. The most comprehensive assessment of α-amylase activity in crustaceans included 40 different species and agreed with the statement above, as revealed that omnivorous crustaceans such as shrimps, crabs and crayfish have relatively high α-amylase activity respect to carnivorous species [18]. However, in other studies the α-amylase activity of some carnivorous crustaceans [19,20] including spiny lobsters [8,11,18] has been observed as high as in herbivorous or omnivorous crustaceans. This high α-amylase activity in spiny lobsters seems also to contradict the limited metabolic use of dietary carbohydrates (e.g., high and prolonged hyperglycemia after feeding) [21,22], probably because reduced activity of enzymes involved in both glycolysis and glycogen synthesis [22].

On the other hand, crustaceans with omnivorous feeding habits including all detritus, plants, and animals in their diet seem to have more α-amylase isoforms than carnivorous, although exceptions occur [18]. Whereas α-amylase polymorphism plays an important role in the variation of biological traits such as feed conversion and growth rate in other taxa [13,23,24], its physiological meaning in crustaceans remains poorly understood. Given the gaps in our understanding of the functional significance of α-amylases in carnivorous crustaceans, we study here the α-amylase enzyme in the spiny lobster, *Panulirus argus*, at the gene, molecular and protein levels. Collectively, our findings shed some light on the gene evolution, source and physiological meaning of polymorphism, and dietary regulation of α-amylase in a carnivorous crustacean.

## Materials and Methods

### Collection of animals and sampling procedure

Lobster (*Panulirus argus*) juveniles were collected in the Gulf of Batabanó, Cuba, by SCUBA diving. The collection area was: 21°39.0431´N—83°09.8436´W; 21°41.0015´N—83°092463´W; 21°40.1016´N—83°11.0297´W, and it was performed under permission of the Fisheries Regulator Department from the Ministry of the Fishing Industry of Cuba. Intermolt animals were placed on ice for 10 min to obtain a chill coma and then were dissected to collect the digestive gland, stomach, intestine, gills, heart and abdominal muscle. Before dissection, hemocytes were collected using citrate/EDTA buffer pH 4.6 as the anticoagulant [25] as describe before [26]. All samples were immediately placed in RNAlater at 4°C for 24 h and then stored at -20°C until use for total RNA extraction. Additional samples of digestive glands were taken for α-amylase zymograms, immediately frozen in liquid nitrogen and then stored at -80°C. Another set of lobsters collected as above were transported alive to the Center for Marine Research of the University of Havana, Cuba, for the feeding trial.

### Classification of individuals by α-amylase isoenzyme pattern (phenotypes)

Substrate (starch)-SDS-PAGE (5% stacking gel, 10% separating gel) was used to determine the composition of α-amylase isoenzyme in the digestive gland as described before [11]. Briefly, samples were neither boiled nor treated with mercaptoethanol before loading into the gel and they were run in a vertical electrophoresis device (Owl P81, 8×10×0.75 cm). Gels were immersed in a starch solution (1%) at pH 6 for 60 min and then stained with iodine/KI solution (10%). Clear bands indicated the presence of α-amylase enzymes. Digestive gland extracts from 126 lobsters were analyzed.

### *In vitro* digestion by the digestion cell method

Digestions were performed using digestion cells [27,28] as modified before [29], for assessing differences in initial rate of digestion among α-amylase phenotypes. This method allows the continuous removal of digestion products and has shown to be sensitive enough for detecting small differences in rate of digestion among isoforms of other digestive enzymes in lobster [29]. Briefly, each digestion cell is composed by an internal reaction chamber formed by a cellulose dialysis membrane with molecular cut off of 1000 Da (Spectra/Por 6, Spectrum Medical Industries, Inc., Los Angeles, CA) fixed within an inverted 50 mL Corning tube that forms an external chamber. A continuous flow of buffer is maintained through the external chamber by a high precision peristaltic pump (Ismatec, Idex Corp.), which allows the constant removal of digestion products while the internal chamber is continually agitated by a magnetic stirrer. Two carbohydrates were selected as substrates (wheat and maize flour) as significant differences in digestibility were found between them in a previous study [22]. Five digestions per phenotype per carbohydrates source (30 digestions) were performed.

The procedure for each digestion was as follows: Carbohydrate sample (1g) was poured into the internal chamber and stirred in a 50 mM sodium-phosphate buffer pH 6.0, 0.1 M NaCl and 20 mM CaCl_2_, at 26°C to achieve a concentration of 6% (w/v). Then, the external chamber was overflowing with the same buffer at 26°C and an individual enzyme extract was added to the internal chamber. At this time a constant flow of buffer (26°C) at a rate of 0.5 mL/min was activated through the external chamber. The amount of extract added for each digestion was adjusted in order to put the same units of α-amylase activity (1 U) in all digestions. Amylase activity in extracts was previously determined using an HELFA Amylase Assay Kit (Quimefa Biologic Products Inc. Havana, Cuba) with CNP-G3 (2-Chloro-4-nitrophenyl-a-D-maltotrioside) as the substrate, following the manufacturer’s instructions. One unit of amylase activity was defined as the amount of enzyme that produces the release of 1 mmol nitrophenol per minute. Dialysates were collected at different times (30 min and 1 to 3 h) for quantifying the amount of glucose released using a HELFA^®^ RapiGluco-Test assay Kit (Quimefa Biologic Products Inc. Havana, Cuba) with CNP-G3 (2-Chloro-4-nitrophenyl-a-D-maltotrioside) as the substrate, following the manufacturer’s instructions. Blank assays without addition of enzyme extract were carried out for each carbohydrate sources.

### Purification of lobster α-amylase

Digestive glands from 25 individuals were pooled and homogenized in water at 4°C with a blender in the presence of diethyldithiocarbamate (0.01 M) to prevent melanization [30]. After centrifugation at 10,000 x *g* for 60 min at 4°C, the surface lipid layer was removed; the supernatant was subjected to 30% ammonium sulfate precipitation for eliminating particulate materials and was centrifuged as above. This supernatant was referred to as clarified crude extract. All chromatographic steps were performed using a GradiFrac system (Pharmacia-LKB, Sweden) coupled to an HPLC pump K-1001 (Knauer, Germany). Seventeen milliliters of clarified crude extract [~111 mg protein mL^-1^ determined by the Lowry method [31], with bovine serum albumin (BSA) as a standard] was fractionated by gel filtration on a Sephadex G-100 column (XK 26–70 Pharmacia) previously equilibrated with 20 mM imidazole pH 6.0 (Buffer A) containing 0.1 M NaCl. The flow rate was 0.5 mL min^-1^, and protein elution was monitored at 280 nm. Fractions of 4 mL were collected for analysis of α-amylase activity using HELFA Amylase Assay Kit (Quimefa Biologic Products Inc. Havana, Cuba) with CNP-G3 (2-Chloro-4-nitrophenyl-a-D maltotrioside) as the substrate, in an ELx808IU microplate reader (BioTek Instruments, Winooski, VT). Those fractions with α-amylase activity were pooled and applied to an anion exchange column (HiTrap^™^ Q HP column 5mL, GE Healthcare Bio-Science) equilibrated in Buffer A with 0.05 M NaCl. The column was washed with 10 mL of the same buffer. Alpha-amylase was eluted with 250 mL of a linear gradient from 0.0 to 1.0 M NaCl in Buffer A at 0.5 mL min^-1^. Fractions of 3 mL were collected for analysis of α-amylase activity as above. During purification α-amylase activity was expressed as ΔAbs min^-1^. A single band under both native-PAGE and SDS-PAGE [32], as well as in the starch zymography [11], was taken as a fraction containing a homogeneous enzyme. The relative molecular weight of α-amylase was determined using SigmaGel v1.0.5.0. (Jandel Scientific) after 12% SDS-PAGE in a P81 Puffin, 8 x 10 x 0.75 cm devise (Owl), using broad range molecular-weight standards (6.5–200 kDa, BIO-RAD).

### Biochemical characterization of lobster α-amylase

For biochemical characterization, α-amylase activity was assessed in a mixture composed of 5 μL of purified α-amylase and 200 μL of assay buffer (50mM MES [2-(N-morpholino) ethanesulfonic acid], pH 5.5, containing 0.5 mM 2-Chloro-4-nitrophenyl-a-D-maltotrioside, CNP-G3) as the substrate. The 2-cloro-4- nitrophenol (CNP) released was measured kinetically at 405 nm for 10 min at 37°C in an ELx808IU microplate reader. Initial velocities were obtained using the kinetic application of the program KC4 version 3.4 (BioTek Instruments). The extinction coefficient of *p*-nitrophenol at 405 nm for a volume of 205 μL was 9.774 mM^-1^ cm ^-1^, as determined empirically.

Using this assay, we first assessed the effect of different NaCl and CaCl_2_ concentrations on the activity of the isolated α-amylase by including in the buffer different concentrations of NaCl (0, 0.025, 0.05, 0.1, 0.2, 0.3, 0.4, 0.5, 1, 1.5, 2 M) and CaCl_2_ (0, 1, 2.5, 6, 12.5, 25, 50 mM). Blanks were prepared using the assay buffer with corresponding NaCl and CaCl_2_ concentrations but without the enzyme. Afterward, 0.3 M NaCl and 25 mM CaCl_2_ were always included in assay buffer. Later, the effect of pH on enzyme activity was evaluated as above but using different buffers (50 mM sodium citrate, pH 2 to 4, 50 mM sodium phosphate, pH 5 to 8, 50 mM glycine, pH 9 to 10).

Also, the effects of different pH and temperatures on the stability of the enzyme were examined by preincubation the lobster α-amylase at different pH (2–10) and temperatures (5–75°C) for 60 min prior to the enzyme assays. Stability results were expressed as residual activity in %. Finally, lobster α-amylase was kinetically characterized using 50 mM MES, 0.3 M NaCl, 25 mM CaCl_2_, pH 5.5 as the assay buffer containing different concentrations of substrate (0.025, 0.05, 0.1, 0.25, 0.5, 1, 1.5, 2 mM of CNP-G3). The kinetic parameters *V*max and *K*m were evaluated by adjusting the data to the Henri-Michaelis-Menten equation. Values of turnover number (*k*cat) were calculated from the following equation: *V*max/[*E*] = *k*cat, where [*E*] is the nominal enzyme concentration. The nominal enzyme concentration was determined at 280 nm using an extinction coefficient (*E*_280_ 1%) (152915 M^-1^ cm ^-1^) deduced from the α-amylase sequence with the ExPASy ProtParam tool (http://web.expasy.org/cgi-bin/protparam/protparam). The software GraphPad Prism ver. 5.0. (GraphPad Software, Inc.) was used for analysis of kinetic data.

### Cloning and sequencing of lobster α-amylase

Alpha-amylase cDNAs from *Oreochromis niloticus* (GenBank acc. no. DQ064646), *Gallus gallus* (NM_001001473), *Penaeus vannamei* (AJ496537), *Drosophila melanogaster* (AY322195), and *Apis mellifera* (AB022908) were retrieved from GenBank/National Center for Biotechnology Information (NCBI) (http://www.ncbi.nlm.nih.gov/), and then ClustalW (http://www.ebi.ac.uk/Tools/msa/clustalw2/) was used to search conserved sequences for designing degenerated primers. The softwares GenRunner v3.05 and Oligo Analyzer v1.1.2 were used for primer analysis. Degenerated primers were synthesized by IDT^®^ (Integrated DNA Technologies) (S1 Table).

Total RNA was isolated from individual digestive glands using an Ultra-Turrax^®^ T25 (IKA^®^-Werke) and the NucleoSpin^®^ RNA II kit (Macherey-Nagel, Düren, Germany), including an on-column RNase-free DNase digestion step. Concentration of total RNA was spectrophotometrically measured at 260 nm with the BioPhotometer Plus (Eppendorf), and its quality was determined in an Agilent 2100 Bioanalyzer (Agilent Technologies, Santa Clara, CA, USA) using the Agilent RNA 6000 Nano Kit. First-strand cDNA was obtained by reverse transcription using 250 ng of random primers (Invitrogen^™^, Life Technologies, Carlsbad, CA, USA) and the Super Script III (Invitrogen). PCR amplification was performed with the first strand of cDNA (corresponding to 100 ng of input total RNA) as template with the high-fidelity proofreading VELOCITY DNA Polymerase (BIOLINE, Berlin-Brandenburg, Germany). Samples were cycled (98°C, 5 min; [98°C, 30 s; 62 to 58.5°C in touchdown, 30 s; 72°C, 1 min] X 35 cycles; 72°C, 10 min) in a Mastercycler^®^proS vapo.protect (Eppendorf, Hamburg, Germany). PCR products were visualized in 1% agarose gel electrophoresis using GelRed^™^ (Biotium) as the stain. PCR products of the expected size were excised from the gel, purified with the NucleoSpin^®^ Gel and PCR Clean-up kit (Macherey-Nagel, Düren, Germany), and cloned on *E*. *coli* (Top 10, Invitrogen^™^) using the pJET1.2/blunt cloning vector of the CloneJET PCR Cloning Kit (Thermo Scientific, Waltham, MA, USA). Several clones for putative α-amylase cDNAs were sequenced in both strands, using pJET1.2 Forward and pJET1.2 Reverse sequencing primers, by the dideoxy method at the Bioarray S.L. sequencing facilities (Alicante, Spain). All kits were used according to manufacturer’s instructions. Sequence homology for all the sequenced PCR products was confirmed by blastn at the NCBI web site (http://blast.ncbi.nlm.nih.gov/Blast.cgi). Fragment assembly was made with the eBiox (v1.5.1) software for Macintosh. Despite the several clones sequenced (24 from eight different degenerated primer combinations) from different individuals *P*. *argus*, this procedure allowed to obtain just a single partial cDNA sequence.

Using total RNA as the template, the 5′ and 3′ ends of α-amylase mRNA were amplified using 5′ and 3′ Rapid Amplification of cDNA Ends (FirstChoice^®^ RLM-RACE kit, Life Technologies^™^). Specific forward primers were designed for the cDNA fragment previously obtained at three different positions (S1 Table) and used in combination with the 3’RACE Outer or Inner primers supplied in the kit to amplify the 3′ ends. For 5′ RACE amplifications, specific reverse primers for the α-amylase cDNA fragment previously obtained were designed (S1 Table) and used in combination with the 5’RACE Outer or Inner primers supplied in the kit. The primers were designed to achieve an overlap of at least 200 bp between the RACE clones and the previously obtained partial cDNA. Cloning, sequencing, and fragment assembly were performed as described above.

### Sequence analysis of lobster α-amylase

The nucleotide sequence of the full-length cDNA obtained was analyzed for homology by blastn at the NCBI website (http://blast.ncbi.nlm.nih.gov/Blast.cgi). eBiox (v1.5.1) software was used for searching polyadenylation sites and for translating the open reading frame (ORF). Homology analysis of the protein sequence was carried out with blastp at the NCBI website. Protein motifs’ were predicted using the Simple Modular Architecture Research Tool (http://smart.embl-heidelberg.de/). Theoretical isoelectric point and relative molecular mass of the deduced protein were predicted using the ExPASy’s Compute pI ⁄Mw tool (http://us.expasy.org/tools/pi_tool.html). Prediction of the signal peptide cleavage site was carried out using SignalP (http://www.cbs.dtu.dk/services/SignalP/). Peptidase cleavage sites in PaAmy sequence were assessed with ExPASy PeptideCutter tool (http://web.expasy.org/peptide_cutter/). *N*-glycosylation and *O*-glycosylation predictions were made using the tools NetNGlyc and NetOGlyc from ExPASy. Further *O*-glycosylation prediction based on surface accessibility was performed with GlycoEP (http://www.imtech.res.in/cgibin/glycoep/glyechk?job=5150&tim=15).

### Tissue distribution of lobster α-amylase by real-time qPCR

Total RNA from equivalent amounts of different organs (digestive gland, stomach, intestine, gills, heart, abdominal muscle, and hemocytes) was purified as described above, and the α-amylase expression assessed by real-time qPCR. Specific primers (S1 Table) were designed using the software Primer3 v.0.4.0 (http://frodo.wi.mit.edu/) for quantification the relative expression of α-amylase and elongation factor 1 alpha (ef1a) as the housekeeping gene [33]. Primers were synthesized by IDT (Integrated DNA Technologies, Leuven, Belgium). Total RNA isolation, quantification and quality assessment were performed as described earlier. Only samples with a RNA Integrity Number (RIN) higher than 8.0 were used. First, total RNA (500 ng) was reverse-transcribed in a 20 μL reaction using the qScript^™^cDNA synthesis kit (Quanta BioSciences). The reaction was performed using qScript ReactionMix and qScript Reverse Transcriptase, with 5 min at 22°C, 30 min at 42°C and 5 min at 85°C. qPCR conditions were optimized by testing different primer concentrations (100 nM, 200 nM and 400 nM) and a temperature gradient from 50 to 60°C. Also, different amounts of cDNA were used in triplicate (6 points of serial 1/5 dilutions from 10 ng to 3.2 pg per reaction) as templates to check the assay linearity (R^2^) and the amplification efficiency (E) of primers. Although the assay was linear along the six serial dilutions (R^2^ = 0.998, E = 0.92), 10 ng of cDNA per reaction was further used in qPCR reactions. qPCR was performed with Fluorescent Quantitative Detection System EppendorfMastercycler^®^ep*realplex*^2^ S, (Eppendorf, Hamburg, Germany). Each reaction mixture (10 μL) contained 0.5 μL at 400 nM of each specific forward and reverse primer, and 5 μL of PerfeCTa SYBR^®^ Green FastMix^™^ (Quanta Biosciences) in white wells twin.tec real-time PCR plates 96 (Eppendorf). Control reactions with DEPC water and RNA instead of cDNA were included to ensure the absence of contamination or genomic DNA. The qPCR thermal profile was: 95°C, 10 min; [95°C, 20 s; 60°C, 35 s] X 40 cycles; melting curve [60°C to 95°C, 20 min], 95°C, 15 s). The melting curve was used to ensure that a single product was amplified and confirm the absence of primer-dimer artifacts. Relative quantification was performed using the 2^ΔΔCT^ method [34]. Additionally, qPCR products were separated on 2% agarose gel and stained with GelRed (Biotium) to evaluate the presence or absence of the cloned cDNA in examined tissues.

### Phylogenetic analysis of lobster α-amylase

Amino acid sequences of α-amylase enzymes from different species were retrieved from the NCBI database. Sequences were aligned and the best-fit model of amino acid substitution was selected by testing alternative models of evolution using both the Akaike information criterion and the Bayesian information criterion implemented in MEGA 6 software [35]. The JTT+G (gamma shape parameter = 0.76) model of evolution was selected for further analysis. Phylogeny was reconstructed by analyzing amino acid sequences of crustacean’s α-amylase using the Neighbor-Joining algorithm of MEGA 6 [35]. Topology robustness was tested with 1000 bootstrap replicates [36].

### PCR amplification of lobster α-amylase genomic sequence

Genomic DNA (gDNA) was isolated from digestive glands (~30 mg) by the salting-out method [37]. gDNA concentration was spectrophotometrically measured at 260 nm with the BioPhotometer Plus (Eppendorf). PCR amplifications were performed using 150 ng of gDNA as template with the high-fidelity proofreading VELOCITY DNA Polymerase (BIOLINE, Berlin-Brandenburg, Germany). Seven pairs of specific forward and reverse primers (S1 Table) were designed according to the lobster α-amylase cDNA at 14 different positions. In choosing primer position, it was taken into account the introns positions in the shrimp α-amylase genes, in order to span putative introns. All 49 possible combinations of primers were tested. PCR reactions were cycled (98°C, 5 min; [98°C, 30 s; 62 to 58.5°C in touchdown, 30 s; 72°C, 1 min] X 35 cycles; 72°C, 10 min) in a Mastercycler^®^proS vapo.protect (Eppendorf, Hamburg, Germany) using all (i.e., 49) possible combination of primers. PCR products were visualized in 1% agarose gel electrophoresis using GelRed (Biotium) as the stain, and those of expected size or larger were cloned and sequenced as described above.

### Three-dimensional (3D) homology modelling of lobster α-amylase

Position-specific iterated blast (psi-blast) (http://blast.ncbi.nlm.nih.gov/Blast.cgi) against the NCBI non-redundant and Protein Data Bank (PBD) databases was used to identify *P*. *argus* α-amylase (PaAmy) related sequences. In the search for structural templates from known structures in the PDB (http://www.rcsb.org/pdb/), we used SWISS-MODEL (http://swissmodel.expasy.org/). Also, we complemented the template SWISS-MODEL predictions with those obtained by phyre (http://www.sbg.bio.ic.ac.uk/phyre2/) [38] and i-tasser (http://zhanglab.ccmb.med.umich.edu/I-TASSER/) [39] in order to gain success in the fold recognition approach. Sequence and 3D structure of human pancreatic α-amylase were retrieved from the UniProt ⁄ Swiss-Prot and the PDB databases, respectively. The profile alignment option of the ClustalX program [40] was used to compare *P*. *argus* and human α-amylase sequences. The aligned sequences were adjusted manually to minimize the number of gaps and insertions. We predicted the PaAmy 3D model using SWISS-MODEL (http://swissmodel.expasy.org/) and human pancreatic α-amylase (PDB: 1B2Y) as the template.

The predicted 3D model for PaAmy was subjected to a series of tests to evaluate its internal consistency and reliability. Backbone conformation was evaluated by the inspection of the Psi ⁄ Phi Ramachandran plot obtained from Procheck analysis [41]. Packing quality of the 3D model was investigated by the calculation of the whatcheck Z-score value [42]. Finally, sequence-structure compatibility was evaluated by verify-3d [43]. All these programs were executed from the structure analysis and verification servers at University of California, Los Angeles (http://www.doe-mbi.ucla.edu/Services/SV/). Protease cleavage sites in PaAmy were identified with the ExPASy PeptideCutter tool and its solvent accessibility was analyzed with I-Tasser [39]. Those cleavage sites predicted to be exposed were then mapped to PaAmy model using the Open-Source PyMOL^™^ Molecular Graphics System, Version 1.6.x (Schrödinger, DeLano Scientific LLC, San Carlos, California, USA).

### Tryptic digestion of lobster α-amylase

Putative proteolysis of purified α-amylase was performed with bovine trypsin as detailed before [44]. Briefly, trypsin-TPCK (mol ratio of trypsin:*α*-amylase = 1:15) was added to a solution of pure α-amylase in 20 mM imidazole, pH 6, containing 20 mM CaCl_2_, and the mixture was incubated at 37^◦^C for 72 h. Aliquots were taken at different reaction times up to 72 h and reactions stopped at -20°C. Additionally, the same procedure was carried out but incubating crude extracts of digestive glands containing α-amylase and all other endogenous digestive enzymes of lobster. Digestions were monitored by SDS-PAGE electrophoresis as described above.

### Periodic acid-Schiff (PAS) staining of lobster α-amylase

Following SDS-PAGE, glycoprotein staining of the purified α-amylase and gastric juice samples of lobsters exhibiting both α-amylase isoforms was performed by PAS staining [45].

### Effects of diet on lobster α-amylase gene expression and activity

Three diets were formulated to have 45% protein, 10% lipids, and 35% of three different carbohydrates (rice starch, wheat flour, maize starch), and referred as rice, wheat, and maize diets (S2 Table). Only apparently healthy intermolt specimens, determined according to [46], were used. Three groups of 5 lobsters were fed during one month with the experimental diets, left unfed for 48 h, and then fed again with the respective diets. They were sacrificed 24 h after last ingestion in an ice-cold water bath and then were dissected to collect gastric juice and digestive glands for enzyme assays. Gastric juice samples were centrifuged at 10 000 x g for 30 min at 4°C, and supernatants were stored at -80°C until used. Digestive gland samples were first homogenized with chilled Milli-Q^®^ water (90 mg/500 μL) using a glass piston homogenizer, and then centrifuged at 10 000 x g for 30 min at 4°C. The resultant upper lipid layers were discarded and the remaining supernatants were stored at -80°C until used. Samples for expression analysis (only digestive glands) were stored in RNAlater as describe before. Five lobsters that were fed during one month with fish flesh were also lead unfed for 48 h, fed and sampled as above, and referred as fresh fish treatment. Alpha-amylase activities were determined using an HELFA^®^Amilase Assay Kit (Quimefa Biologic Products Inc. Havana, Cuba). One unit of α-amylase activity was defined as the amount of enzyme that produces the release of 1 μmol nitrophenol per minute. Units of α-amylase activity were expressed per volume of gastric juice or weigh of digestive gland. Gene expression was quantified by real-time qPCR as detailed above.

### Statistical analyses

Only results from intermolt lobsters were analyzed as molt stage has been found to affect digestive enzyme activities [47] and feeding activity [48] in *P*. *argus*. All data were checked for normality and homogeneity of variance using D'Agostino-Pearson and Levene’s tests, respectively, with P<0.05. The progress of *in vitro* digestions was modeled by linear regressions obtained by the least-squares method. The significance of regression slopes were tested by ANOVA (P<0.05) and R^2^ were calculated as a measure of relative goodness of fit of regression curves. Differences among initial rates of digestion (slopes) were assessed with ANCOVA by making all pairwise contrasts with the Bonferroni adjustment of significance levels to correct for multiple testing [49]. Alpha-amylase activity in digestive gland and gastric juice, and α-amylase expression in digestive gland (N = 5 lobsters, per dietary treatment) were analyzed by one-way ANOVA (P<0.05), being the experimental diets the source of variation. In all cases, the Tukey’s test (P<0.05) was used to determine differences among means. The software package Statistica 7.0 (StatSoft Inc., Tulsa, OK, USA) was used for all tests performed. Graphs were generated by GraphPad Prism 5.00 (GraphPad Software, Inc., San Diego, California, US).

## Results

### Most frequent α-amylase phenotype in lobster has the lowest carbohydrate digestion efficiency

**Fig 1:**
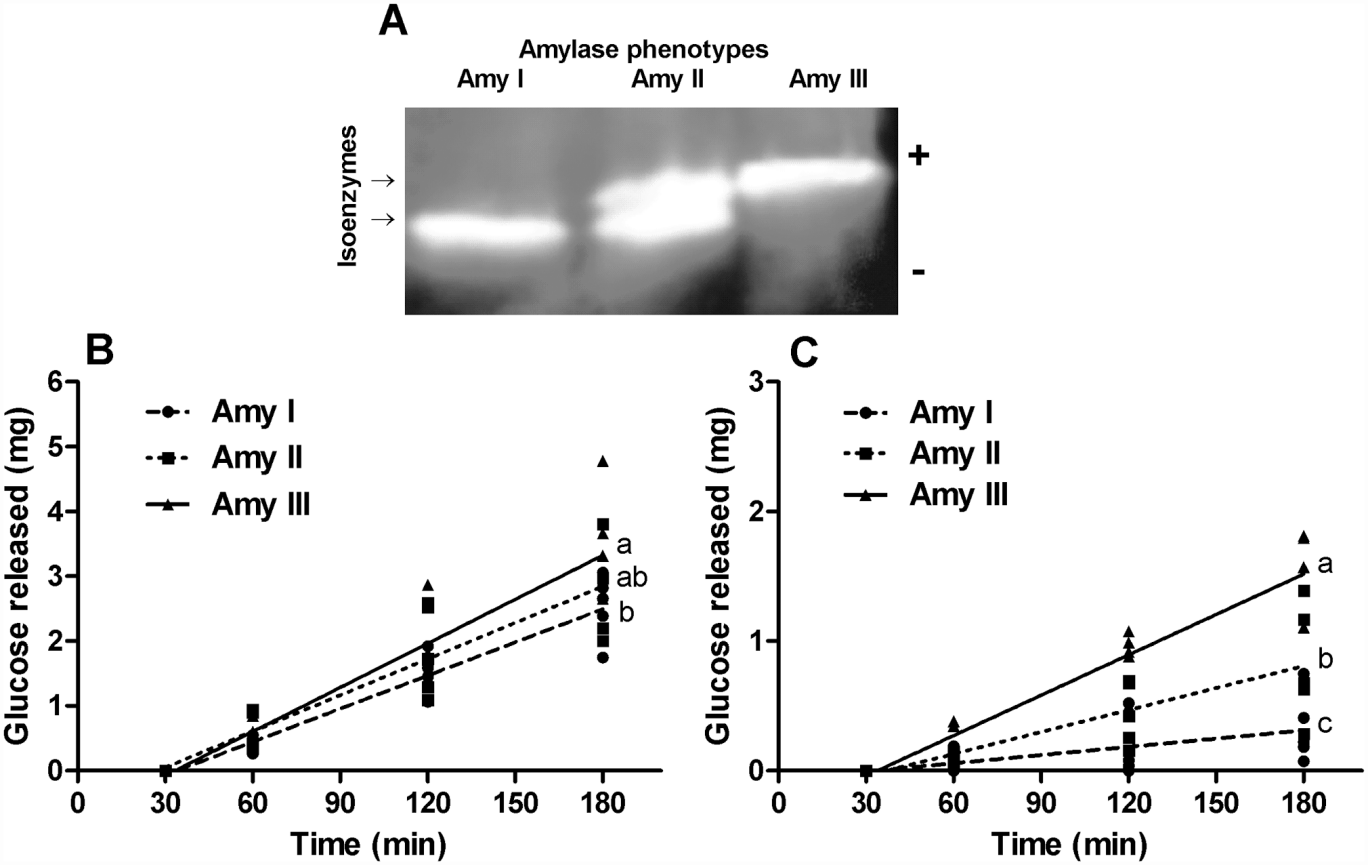
Alpha-amylase phenotypes in the lobster *Panulirus argus* differ in digestion efficiency. A) Alpha-amylase isoenzyme patterns (i.e., phenotypes) in lobster revealed by starch zymography. Lobsters with the isoenzyme of higher electrophoretic mobility are named phenotype Amy I, individuals with both isoenzymes are named phenotype Amy II, and lobsters with the isoenzyme of lower electrophoretic mobility are named phenotype Amy III. Graphs represent the kinetic of glucose released (cumulative values against time) from wheat flour (B) and maize starch (C) during *in vitro* digestion by crude digestive extracts of *P*. *argus* with the different α-amylase phenotypes. Letters to the right of regression lines indicate differences (P<0.05) among slopes.

We first determined by substrate (starch)-SDS-PAGE the distribution of the two main starch-degrading enzymes and the resultant phenotypes in the digestive gland of 126 individual lobsters. Individuals with the isoenzyme of higher electrophoretic mobility (44 kDa) were named phenotype Amy I. Lobsters with the two isoenzymes were named phenotype Amy II, and lobster with the isoenzyme of lower electrophoretic mobility (47 kDa) were named phenotype Amy III (Fig 1A). Most lobsters exhibited the phenotype Amy I (62.7%) followed by the phenotype Amy II (28.6%). Lobsters with the phenotype Amy III were the less common (8.7%).

Later, we examined *in vitro* whether the three α-amylase phenotypes differed in digestion efficiency using the digestion cell method. The kinetics of glucose release from digestion cells was best described by linear regressions, all of them with high determination coefficients (R^2^ = 0.73 to 0.96), whose slopes (i.e., rate of digestion) were compared by ANCOVA (P<0.05). We found higher rates of digestion for the wheat flour than for the maize starch, irrespective of the α-amylase phenotype (Fig 1B and 1C). However, for each carbohydrate substrate, we observed differences among phenotypes in their digestion efficiency. Hydrolysis rate of wheat flour by phenotype Amy III resulted significantly higher than by Amy I, while Amy II digested this carbohydrate at an intermediate rate (Fig 1B). Differences among phenotypes were more evident for the less digestible substrate (i.e., maize starch), for which digestion efficiency were: Amy III > Amy II > Amy I (Fig 1C). Control digestions without the addition of enzyme extracts showed negligible release of glucose supporting that the responses observed were due to the hydrolytic activity of carbohydrases in the extracts. Although carbohydrate hydrolysis is the result of the coordinated action of different carbohydrases in addition to α-amylase, the other enzymes were assumed to be homogeneously distributed among extracts.

### A single α-amylase was purified and characterized from lobster

**Fig 2:**
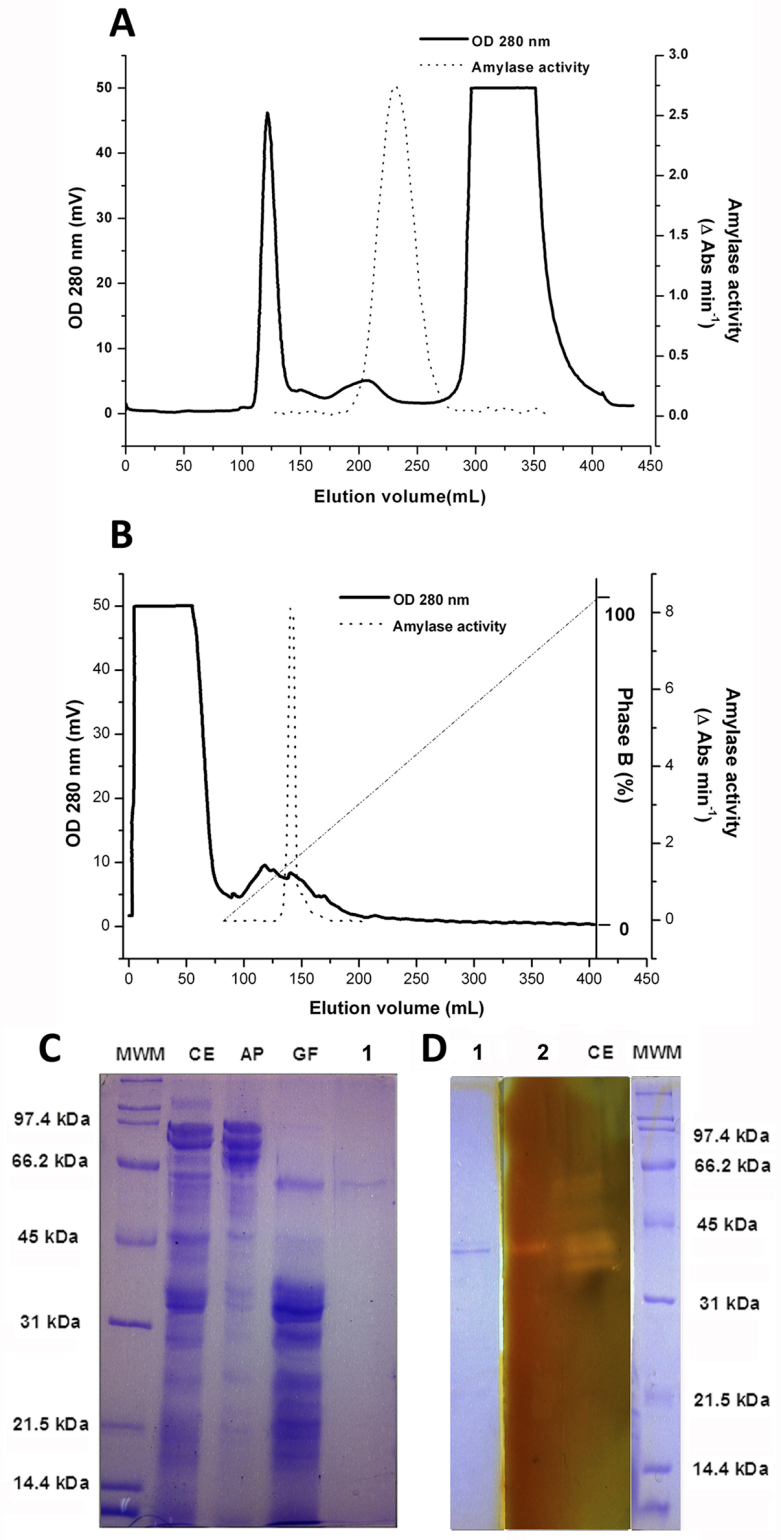
Purification of the lobster *Panulirus argus* α-amylase. A) Gel filtration chromatography profile. Seventeen milliliters of clarified crude extract (~111 mg/ml) obtained from digestive gland of 30 lobster was fractionated by gel filtration on a Sephadex G-100 column (2.6 X 66 cm) previously equilibrated with 20 mM imidazole pH 6.0, 0.1 M NaCl. Fractions of higher α-amylase activity (175–225 ml) were pooled and used for further purification step. B) Anion-exchange chromatography. The pooled fractions from Sephadex G-100 chromatography were applied to a HiTrap column 5 mL, α-amylase isoform was separated with 250 mL of a linear gradient from 0.0 to 1.0 M NaCl. The flow rate was 0.5 mL/min, and protein elution was monitored at 280 nm. Fractions of different step were collected, and measured for α-amylase activity using 2-Chloro-4-nitrophenyl-a-D maltotrioside (CNP-G3) as substrate (dashed line). C) 10% SDS-PAGE of MWM: molecular weight markers, CE: crude extract, AP: ammonium sulfate precipitation, GF: gel filtration, PaAmy: isolated *P*. *argus* α-amylase after anion-exchange. D) lane 1: 10% Native-PAGE of PaAmy and lane 2: starch zymogram of the isolated enzyme.

Our findings on differences in digestion efficiency among α-amylase phenotypes suggested that the two isoenzymes differed in some functional properties, such as less activity in the most frequent isoenzyme. To corroborate this hypothesis we attempted to isolate the two form of the enzyme for further characterization, starting from a crude extract containing both isoforms as judge by starch zymogram. We used gel filtration on Sephadex G-100 followed by anion exchange chromatography on HiTrap^™^ Q HP, and failed in isolating the two enzymes. Alpha-amylase activity eluted between two major protein peaks in the gel filtration chromatography (Fig 2A), leading to a 73-fold purification (Table 1). Fractions with α-amylase activity from gel filtration were pooled and further purified up to 1196.58 folds by anion exchange chromatography (Table 1). Only one peak with α-amylase activity was observed during anion exchange chromatography (Fig 2B), and subsequent chromatographies with a variety of gradients produced the same result. The combined analysis of this peak by SDS-PAGE (Fig 2C), Native-PAGE (Fig 2D, lane 1) and starch zymography (Fig 2D, line 2), revealed that a single lobster enzyme, with apparent molecular mass of 60 kDa (Fig 2D), was purified to homogeneity.

**Table 1:** Purification of α-amylase from the lobster *Panulirus argus*.

Step	Volume	Total protein (mg)	Total activity (ΔOD/min)	Specific activity (ΔOD/min/mg)	Yield	Fold (purification factor)
**Crude extract**	40	10728.00	429.12	0.04	100.00	1.00
**(NH4)2SO4 precipitation (30–60%)**	17	1887.00	314.57	0.17	73.31	4.17
**Sephadex G100**	88	69.85	204.57	2.93	47.67	73.22
**5mL HiTrap (PaAmy)**	9	0.20	9.56	47.86	2.23	1196.58

**Fig 3:**
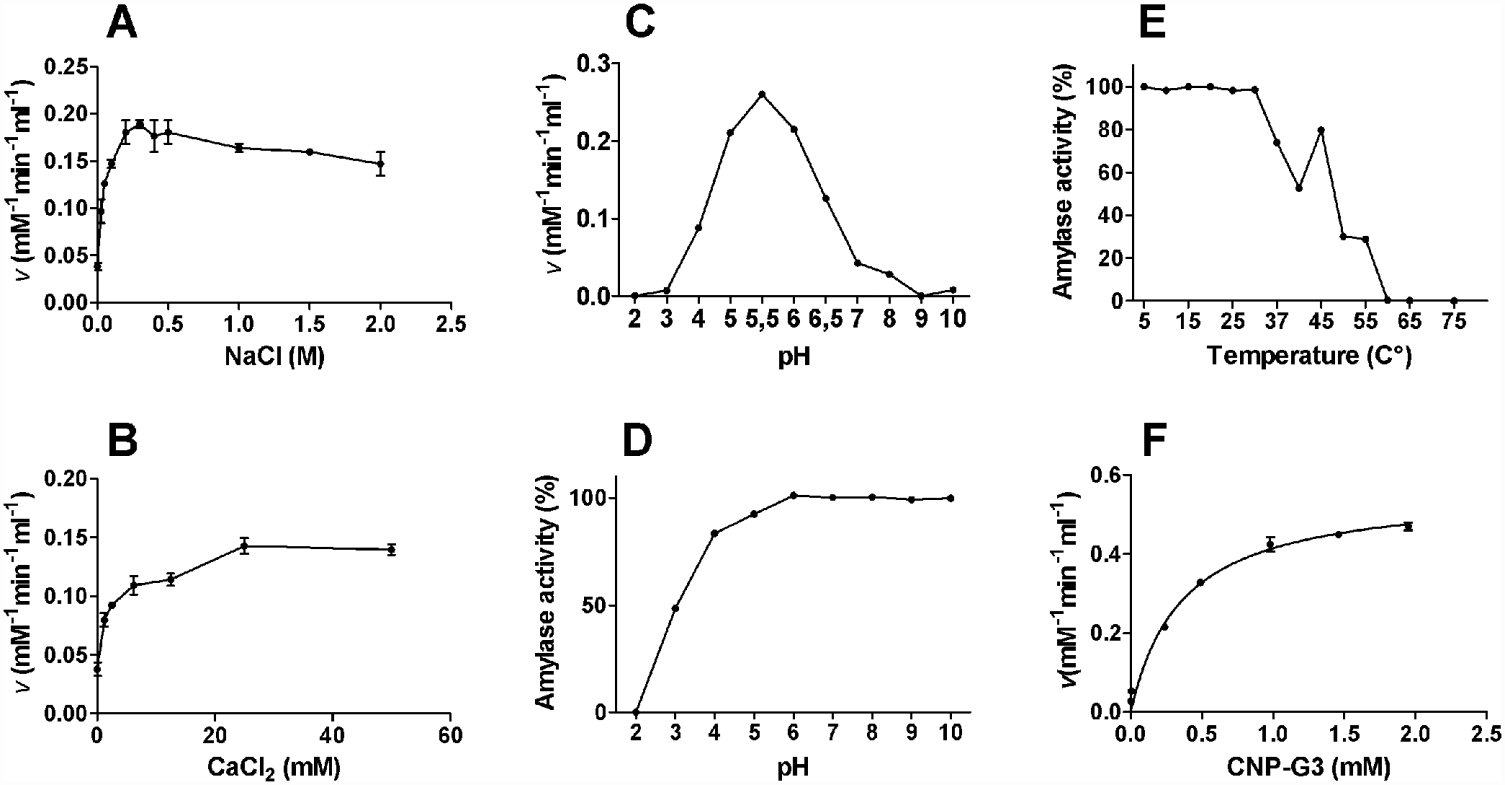
Biochemical characterization of α-amylase from the lobster *Panulirus argus*. Effect of NaCl (A), CaCl_2_ (B), pH (C) on the α-amylase activity and stability of the enzyme under different pH (D) and temperature (E). The enzyme activity was measured using 0.5 mM 2-Chloro-4-nitrophenyl-a-D maltotrioside (CNP-G3) as the substrate in phosphate buffer (pH 5.5, except in the pH effect assays) containing 0.3 M NaCl and 25 mM CaCl_2_ at 37°C. F) Henri-Michaelis-Menten plot of *Panulirus argus* α-amylase (R^2^ = 0.9851) with CNP-G3 as the substrate. The nominal concentration of *P*. *argus* α-amylase was 152915 M^-1^ cm ^-1^. The *K*m and *kcat* values of the enzyme are 0.36 ± 0.052 mM mL^-1^ min^-1^ and 0.56 ± 0.024 mM mL^-1^ min^-1^, respectively.

The activity of lobster α-amylase increased with ionic strength up to 0.3 mM NaCl, and slightly declined at higher NaCl concentrations (Fig 3A). Alpha-amylase activity also rose as CaCl_2_ increased up to 25 mM, but remained unaffected at higher CaCl_2_ concentrations (Fig 3B). The enzyme optimal pH was 5.5 (Fig 3C), whereas poor stability at acidic and relatively high stability at alkaline pH were found (Fig 3D). Stability of the enzyme was compromised at the long-term above 30°C (Fig 3E). Using CNP-G3 as the substrate, we determined that the lobster α-amylase has *K*m of 0.36 ± 0.052 mM, *V*max of 0.56 ± 0.024 mM mL^-1^ min^-1^, and *kcat* of 28.42 ± 1.203 s^-1^ (Fig 3F).

### Lobster expresses a single and highly conserved digestive α-amylase

**Fig 4:**
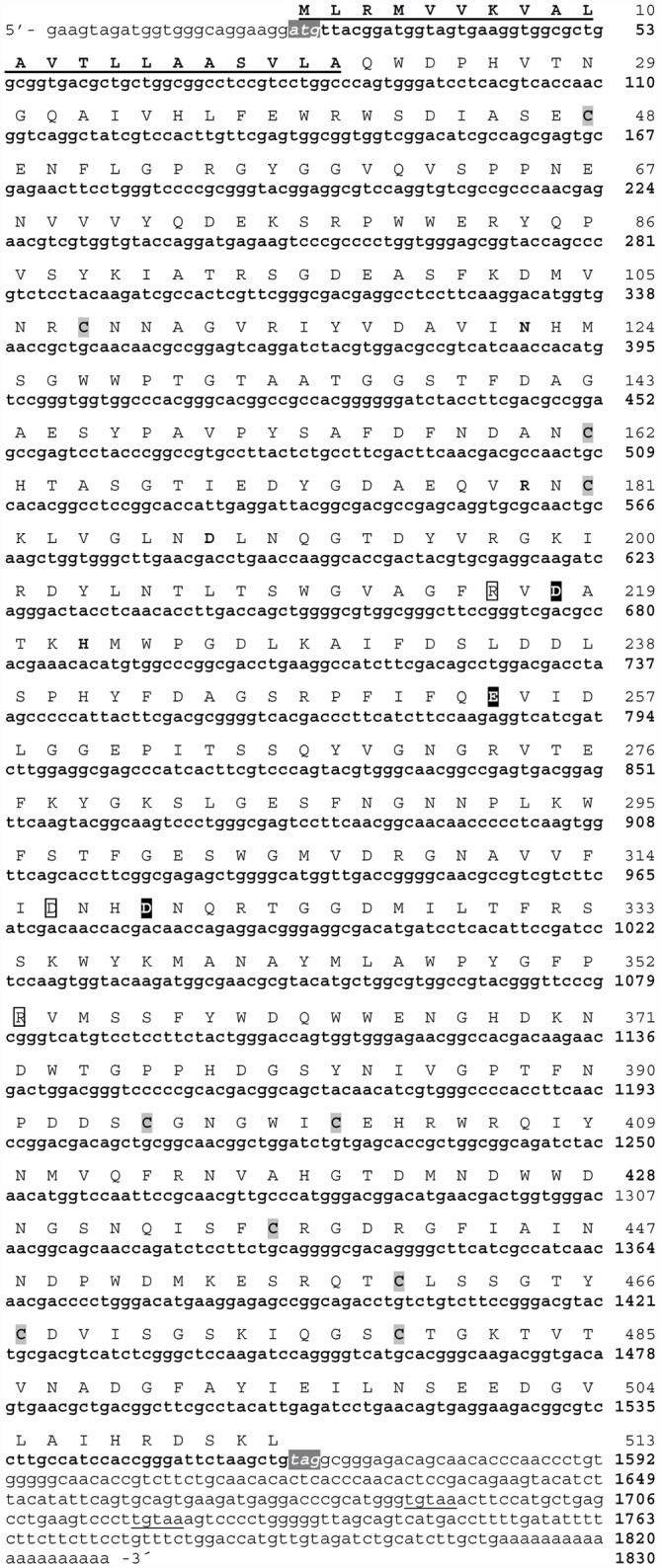
Nucleotide cDNA and predicted amino acid sequences of *Panulirus argus* α-amylase (PaAmy). The start (ATG) and stop (TAA) codons are shaded in dark gray. A putative signal peptide is underlined. The predicted amino acid sequence is displayed above the nucleotide sequence, indicating the open reading frame (ORF). Cysteine residues are shaded in light gray. Active site residues (Asp218, Glu 254 and Asp319) are shaded black. The residues involved in calcium binding (Asn122, Arg179, Asp188, and His222) are shown in bold, and the residues of the chloride binding site (Arg216, Asn317, Arg353) are shown in line box. Two sites of alternative polyadenylation are underlined, with the poly A tail at the end.

We next asked whether the two form of α-amylase in lobster can be explained by different transcripts, giving rise to highly similar proteins that cannot be resolved by the purification methods used. Using degenerated primers we cloned and then sequenced several cDNA fragments from digestive glands of lobster with one or the two isoenzymes. After assemblage of fragments, only one partial transcript could be identified. No other transcript arose due to variation in the 5´ UTR and 3´ UTR when we obtained these ends by RACE, thus we only found one full-length cDNA. This sequence (PaAmy, GenBank accession no. LK937698) was 1830 bp long, with a short 5´ untranslated region of 23 bp, a long 3´ untranslated region of 268 bp, and a 1539 bp ORF (Fig 4). Before the poly A tail, two sites of alternative polyadenylation were found at 108 bp and 139 bp downstream the stop codon (Fig 4). PaAmy cDNA sequence exhibited high identity with *L*. *vannamei* (79%) and *M*. *japonicus* (78%) α-amylase cDNAs (Table 2).

**Table 2:** Identity analysis of *Panulirus argus* α-amylase (PaAmy) protein sequence with α-amylases from other species.

Species	Accession number	Identity (%)	Amino acids
*Litopenaeus vannamei*	CAA54524	79	512
*Marsupenaeus japonicus*	AHN91844	78	512
*Daphnia pulex*	EFX81580	63	513
*Tribolium castaneum*	AGW27506	55	490
*Drosophila melanogaster*	BAB32533	53	494
*Apis mellifer*	BAA86909	53	493
*Blattella germanica*	ABC68516	59	515
*Tenebrio molitor*	P56634	56	471
*Anguilla japonica*	BAB85635	55	512
*Haplochromis burtoni*	XP 005924697	60	512
*Oryzias latipes*	XP_004085115	58	512
*Salmo salar*	ABD13895	56	505
*Tetraodon nigroviridis*	AJ427289	59	512
*Pagrus major*	BAL14133	56	512
*Siniperca chuatsi*	ACJ06746	57	512
*Xenopus laevis*	BC056841	57	511
*Gallus gallus*	AAC60246	60	512
*Meleagris gallopavo*	XP 003208696	60	512
*Cavia porcellus*	XP 005007889	60	511
*Physeter catodon*	XP 007185800	60	511
*Turcius truncatus*	XP 004320419	59	511
*Loxodonta africana*	XP 003409577	61	511
*Trichechus manatus*	XP 004320419	60	511
*Pan paniscus*	ABW02892	59	511
*Homo sapiens*	AAA51724	61	511

**Fig 5:**
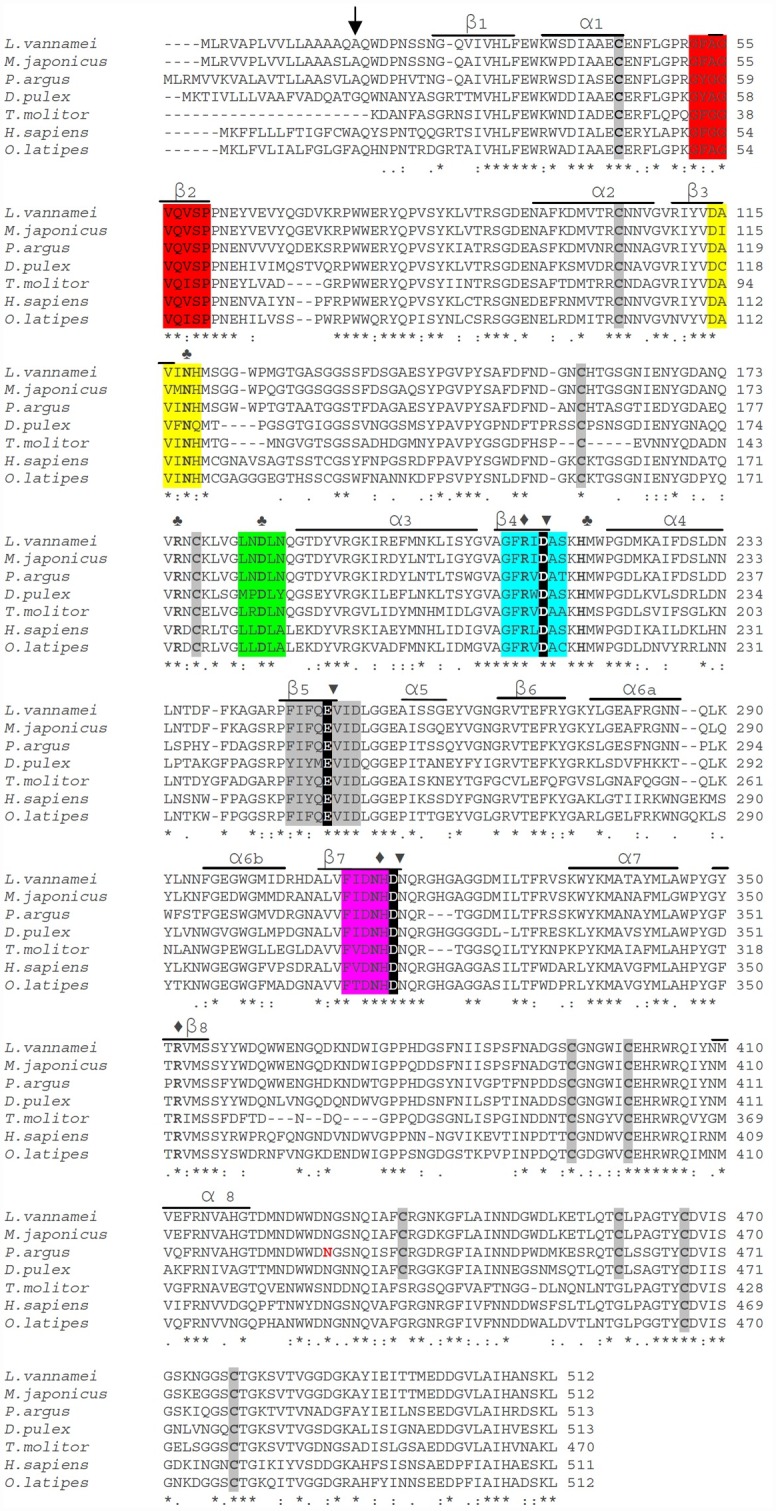
Conserved of amino acid sequence of *Panulirus argus* α-amylase respect to other known α-amylases (*L*. *vannamei*, accession no. CAA54524, *M*. *japonicus*, AHN91844, *Daphnia pulex*, EFX81580, *Tenebrio molitor*, P56634, *H*. *sapiens*, AAA51724 and *Oryzias latipes*, XP_004085115). Identical residues in all sequences are indicated by (*) under the column, conserved substitutions are indicated by (:), and semi-conserved substitutions are indicated by (.). Deletions are indicated by dashes. The predicted peptide cleavage site is indicated by an arrow. Cysteine residues are shaded in light gray. Active site residues are shaded black and labeled (▼). Calcium binding residues (Asn122, Arg179, Asp188, and His222) are shown in bold and labeled (♣), and chloride binding site residues (Arg216, Asn317, Arg353) are shown in bold and labeled (♦). Positions of β-sheets and α-helices are indicated by lines over the sequence. Known conserved sequence regions in the alpha-amylase family: region VI in red, region I in yellow, region V in green, region II in blue, region III in grey, region IV in pink. The less conserved seventh region was not identified in β8. Asparagine predicted to be *N*-glycosylated is highlighted in red.

We obtained additional identity confirmation from the analysis of the encoded protein. The PaAmy transcript encodes a protein with 513 amino acids, including a highly hydrophobic signal peptide of 21 amino acids, a potential cleavage site for the signal peptide between A21 and Q22 (Fig 5), and predicted molecular mass and isoelectric point for the mature enzyme of 55.5 kDa and 4.93, respectively. Pair-wise amino acid alignment revealed that the lobster protein shares a high amino acid conservation respect to other α-amylases (Fig 5). High conservation in α-amylases in crustaceans was also revealed by a phylogenetic analysis, whose topology resembles phylogenetic relationship among groups. Within the well-supported Arthropoda clade, the lobster enzyme appeared in a monophyletic group with α-amylases from other crustaceans (S1 Fig). The lobster α-amylase we described here appeared to be functional, as all typical structural and functional elements of α-amylases could be identified in its sequence (Fig 5). Also, we found by qPCR no expression of PaAmy in haemocytes, gills, heart and muscle, nor in digestive tissues (i.e. stomach, intestine) other than the digestive gland (S2 Fig), thus confirming its exclusive digestive function.

### Gene and intron loss have occurred through evolution of lobster α-amylase

Given that we failed in demonstrating that digestive α-amylase isoforms in lobster are encoded by different transcripts, and that at least three different genes have been reported in other crustacean such as shrimp, we searched in genomic DNA (gDNA) for different genes that would transcribe the same messenger but differed to some extent in intronic sequences. In spite of the several pair of specific primers we used and their different positions (S1 Table), we always retrieved the same genomic sequence after intense cloning and sequencing, suggesting that in contrast to shrimp, a single gene occurs in lobster. Strikingly, we found no intronic sequence within the lobster gene. Several primers were designed in order to span in the lobster DNA the equivalent regions that in shrimp contain one or more introns, but we always obtained fragments that exactly matched the cDNA previously described (Fig 4). The facts that no reverse transcription was done, and that samples were previously treated with RNases, ensured no RNA/cDNA in our starting material.

### Three-dimensional structure of lobster α-amylase

We built a three-dimensional (3D) model for the lobster enzyme (PaAmy) by homology modelling using the x-ray resolved human pancreatic α-amylase (PDB: 1B2Y) as the template. Sequence identity between these two proteins was 60.9%, and according to most of the prediction methods used, this template ranked highest. The PaAmy model obtained was subjected to extensive validation analysis (Table 3). The stereochemical quality of the model was assessed by analyzing residue-by-residue geometry and overall structure geometry with procheck. The PaAmy model has 83.7% residues in most favored regions, 99% of residues in more favored and additional allowed regions together, and only three residues (S77, S431, and V385) in generously allowed regions (Table 3). Only W127 appeared in a disallowed region (0.2%, Table 3). In any case, this last modelling artifact lies well below an acceptable level of 0.5–1%. Additionally, analysis of PaAmy packing quality with whatcheck revealed a good (-0.7) Ramachandran plot Z-score (Table 3). Sequence-structure compatibility was assessed with verify-3d and passed with 97.8% of residues having 3D-1D score > 0.2 (Table 3). In general, PaAmy model scores are similar to those observed in the template structure (Table 3) thus no significant shortcomings are noticeable in the model. PaAmy model was deposited at the Protein Model Data Base (http://bioinformatics.cineca.it/PMDB/main.php) under PMDB id: PM0079556.

**Table 3:** Structural validation of *Panulirus argus* α-amylase (PaAmy) model.

	Template / Model	
Validation criteria	1B2Y	PaAmy
**Procheck**		
Most favored regions	88.1%	83.7%
Additional allowed	11.4%	15.3%
Generously allowed	0.5%	0.7%
Disallowed regions	0.0%	0.2%
**Whatcheck (Z-scores)**		
Ramachandran plot appearance	-0.523	-0.702
Second generation packing quality	-1.863	-2.765
chi-1/chi-2 rotamer normality	-1.073	-1.041
Backbone conformation	-0.775	-1.175
RMS Z-scores, Bond lengths	0.436	0.756
RMS Z-scores, Bond angles	0.768	1.126
Omega angle restraints	0.308	1.015
Side chain planarity	0.586	1.742
RMS Z-scores, Improper dihedral distribution	0.930	1.689
**Verify 3D**		
3D-1D score (>0.2)	99.6%	97.8%

**Fig 6:**
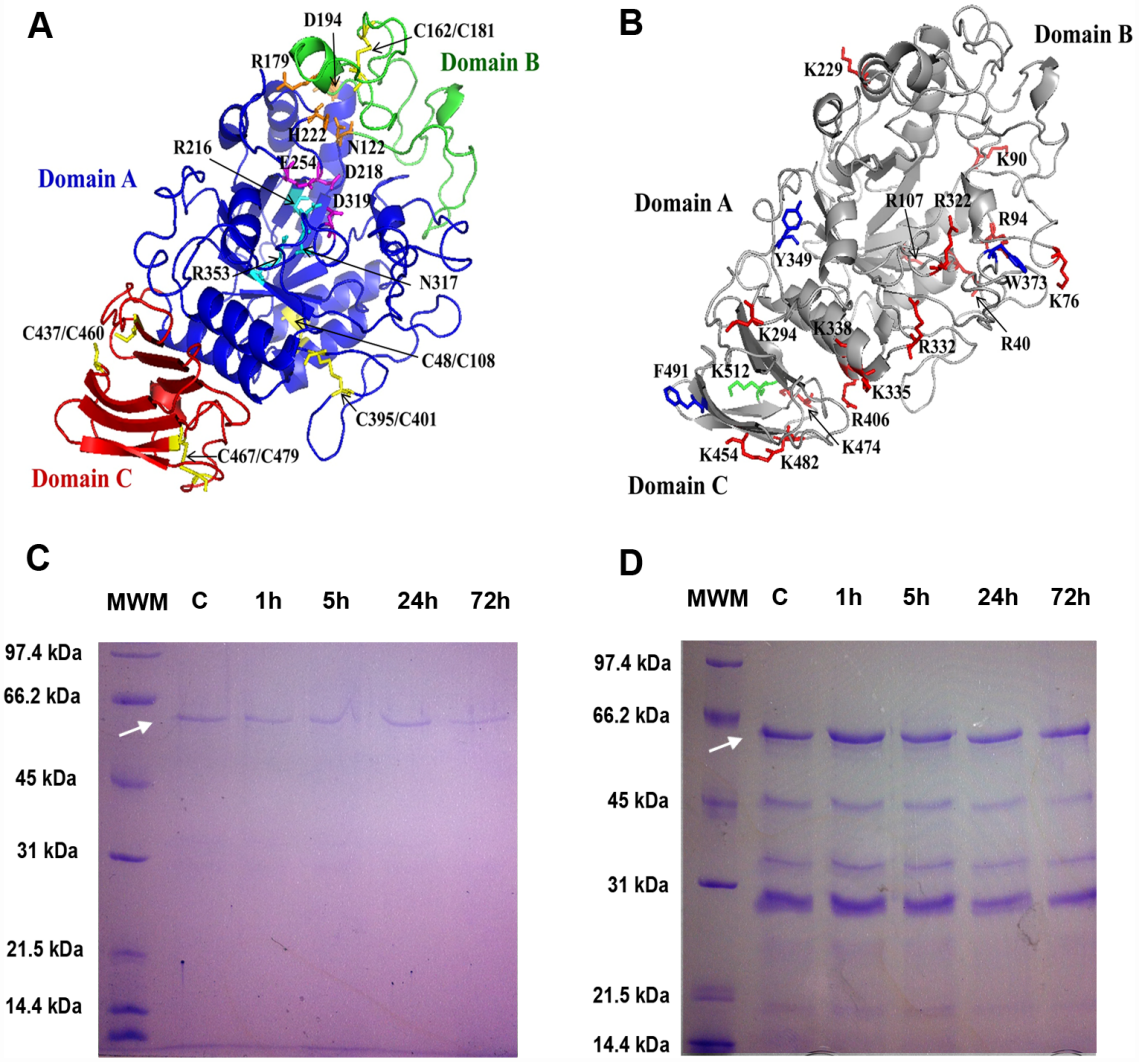
Overall three-dimensional structure of *Panulirus argus* α-amylase as predicted by homology modeling and proteolytic stability of the enzyme. A) Individual domains and key structural and functional residues represented in the model (PMDB database: accession number PM0079556). Domain A (the catalytic domain) is shown in blue, domain B in green, and domain C in red. Residues of the calcium and chloride binding sites are represented in orange and cyan, respectively. Residues of the catalytic triad are depicted in magenta. Cysteines involved in disulfide bridges are highlighted in yellow. B) Putative peptidases cleavage sites in PaAmy mapped to the model. Only cleavage sites exposed to solvent are shown. Cleavage sites for trypsin, chymotrypsin, and carboxypeptidase B are shown in red, blue, and green, respectively. Site numbers start at the first residue of PaAmy, including a 21 residues signal peptide not included in the model. Model was built using SWISS-MODEL (http://swissmodel.expasy.org/) and the human pancreatic α-amylase (PDB: 1B2Y) as the template. Figures were drawn using PYMOL (http://www.pymol.org/). C) Proteolytic stability of isolated *P*. *argus* α-amylase. The isolated enzyme was incubated for 72 hours with the bovine trypsin at a ratio 1:15 (trypsin: α-amylase) and checked by SDS-PAGE at different intervals. MW: molecular weight marker, C is the control (pure α-amylase without bovine trypsin), and the remaining lanes show the time of incubation with trypsin. D) The same procedure as in C but incubating up to 72 h crude extracts of digestive glands containing α-amylase and all other endogenous digestive enzymes of lobster. The arrow indicates α-amylase.

The enzyme has the typical 3D structure of α-amylase enzymes. It is formed by three domains A, B, C. Domain A is a (β/α)_8_-barrel, B is a loop between the β3 strand and α3 helix of A, and C is the C-terminal extension (Fig 6A). PaAmy has the active site cleft between domains A and B, where a triad of catalytic residues (D218, E254 and D319) was found. Other important structural features of α-amylase enzymes such as the calcium (N122, R179, D188, and H222) and chloride (R216, N317, and R353) binding sites were also observed (Fig 6A). Four of the five disulfide bridges found in vertebrates α-amylases were conserved in PaAmy (Figs 5 and 6A), and two additional cysteine residues within domain C may be engaged in a fifth disulfide bridge (Figs 5 and 6A).

### Lobster α-amylase polymorphism arises by glycosylation instead of by limited proteolysis

**Fig 7:**
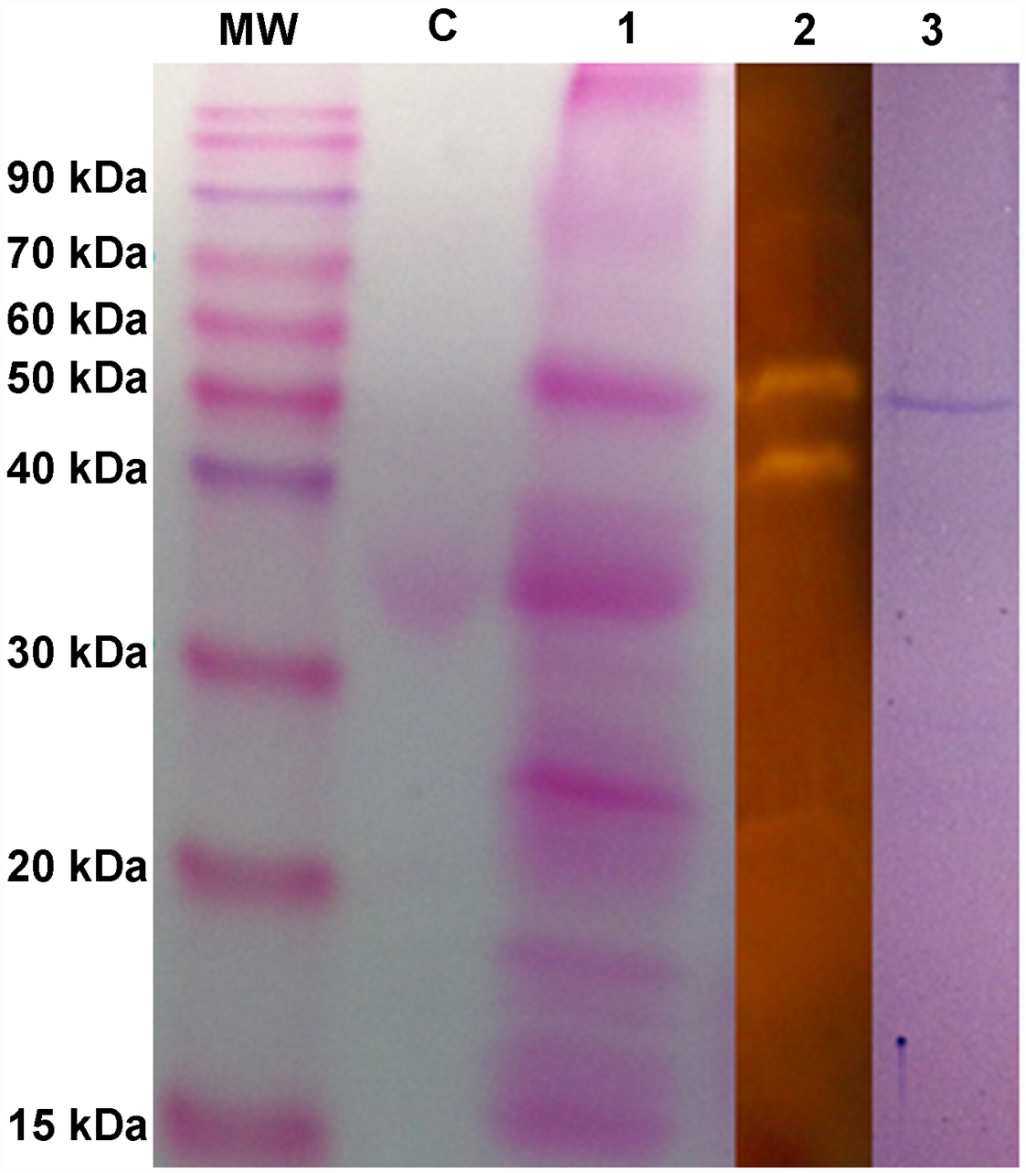
Periodic acid-Schiff (PAS) staining of lobster α-amylase. Glycoprotein staining of purified α-amylase and gastric juice samples of lobsters exhibiting both α-amylase isoforms after 10% SDS-PAGE. Samples were not boiled nor reduced before loaded into the gel. MWM: molecular weight markers, C: glycosylated control protein ovoalbumin, lane 1: gastric juice of lobster containing the main two isoforms, lane 2: starch zymograms of gastric juice of the same lobster, lane 3: purified lobster α-amylase isoform.

We hypothesized that α-amylase polymorphism in lobster could arise by limited proteolysis of a single gene product. We first used our 3D model to predict putative cleavage sites for different digestive proteases. Several cleavage sites were found for trypsin, but also for chymotrypsin, and carboxypeptidase B. From these sites, those that lie in the PaAmy surface were mapped on the PaAmy 3D model and putative trypsin cleavage sites were the most abundant (Fig 6B). However, we could not observe the appearance new bands in SDS-electrophoresis when the purified lobster α-amylase was incubated with the bovine trypsin for 72 hours (Fig 6C) nor when crude extracts of the gland where incubated overnight at room temperature or 37°C (Fig 6D). Thus, other post-translational modifications may be involved in α-amylase polymorphism in lobster. By using the tools NetNGlyc and NetOGlyc from ExPASy, we predicted one (N429) site for *N*-glycosylation and several sites for *O*-glycosylation in the lobster enzyme. However, when surface accessibility was taken into account using GlycoEP, no *O*-glycosylation was predicted for this protein. Further PAS staining of the purified enzyme and of proteins in gastric juice of lobsters exhibiting both α-amylase isoforms showed that the purified isoform (the slower migrating form) is glycosylated whereas the other does not (Fig 7).

### Lobster α-amylase is regulated by diet at the expression level

**Fig 8:**
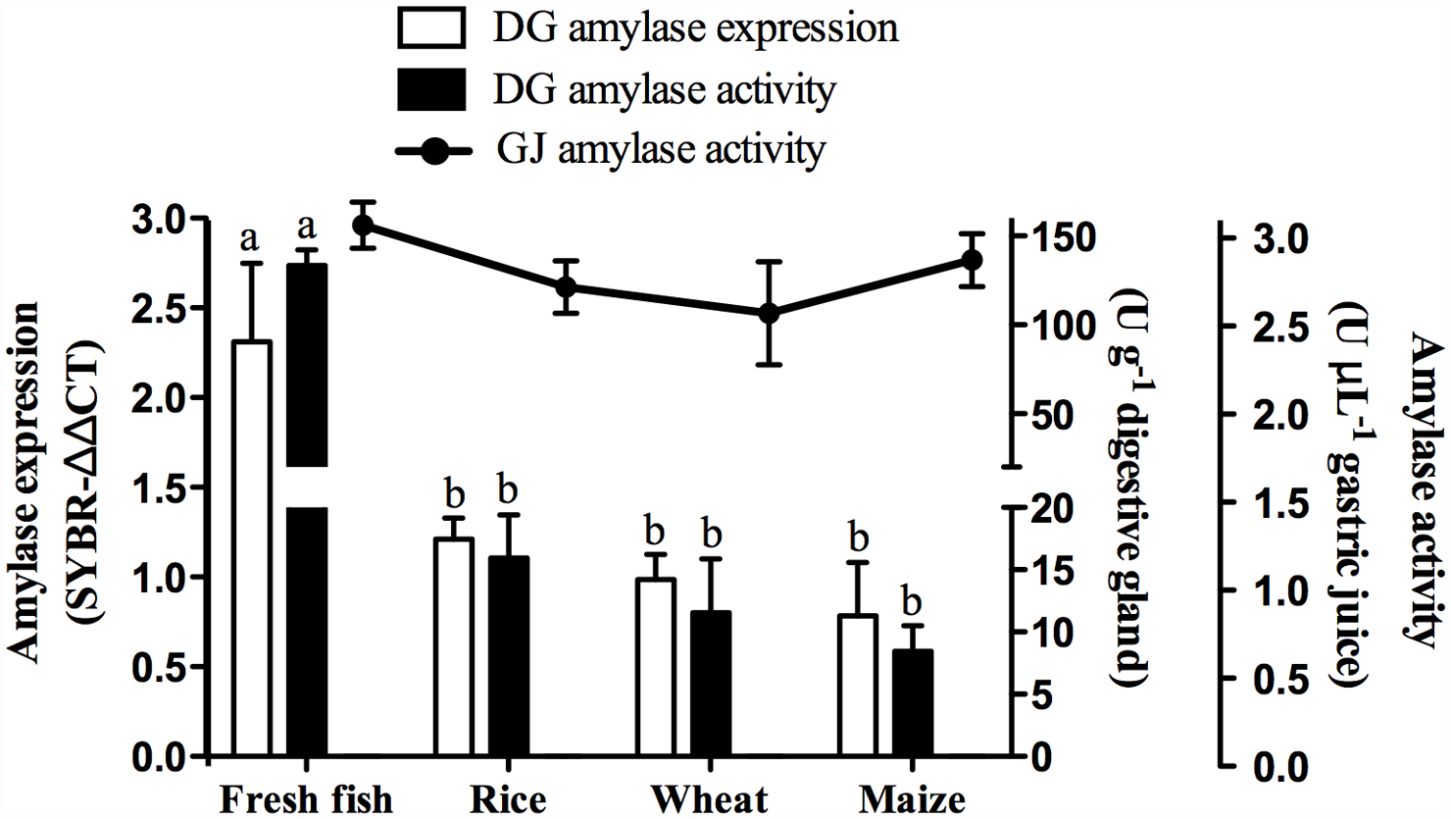
Alpha-amylase in the lobster *Panulirus argus* is regulated by diet at the transcription level in the digestive gland. Alpha-amylase activity and gene expression in the digestive gland (DG) of *P*. *argus* (*N* = 5) 24 h after ingestion different formulated diets or fresh fish. Rice, Wheat and Maize refer to the major carbohydrate source in formulated diets. Values are means + s.e.m. Different letters above the bars indicate statistical differences according to the Tukey’s test (*P*<0.05). Differences were found (*P*<0.05) in α-amylase expression and activity in the digestive gland, but no differences in activity were found in the gastric juice.

Studies in crustaceans with omnivorous habits have revealed a more complex gene, transcript and isoenzyme scenarios than the one we depicted here for the carnivorous lobster. Thus, we next sought to establish whether α-amylase in lobster is tightly regulated by diet or if its regulation has been also simplified through evolution. We fed lobster with fresh fish or three formulated diet containing similar amount of starch, and examined α-amylase expression and activity 24 h later. Gene expression was assessed by real-time qPCR using elongation factor 1 alpha (*ef1a*) as the reference gene, as it showed low variability (less than 0.21 Ct) among experimental groups. Twenty-four hours after ingestion, lobsters fed with fresh fish had the highest expression level of PaAmy (One-way ANOVA, F = 6.15, P<0.05, Tukey’s test, P<0.05) (Fig 8) and the highest α-amylase activity in the digestive gland (One-way ANOVA, F = 133.4, P<0.001, Tukey’s test, P<0.05), while no differences in expression and activity were found in animals fed with the three formulated diets (Fig 8). However, we noticed that lobsters from all dietary treatments presented equally high α-amylase activity in the gastric juice (One-way ANOVA, F = 1.45, P>0.05) (Fig 8).

## Discussion

Alpha-amylases have proven to constitute good models for studying adaptive evolution in different groups such as mammals [15] and insects [50], as interact with exogenous substrates from the environment. In this sense, crustaceans offer a wide platform for advancing the understanding of adaptive digestive physiology as they include species with remarkably different feeding habits from mostly herbivorous to strict carnivorous. However, molecular information on α-amylase is restricted to few omnivorous species such as shrimps [51].

We previously reported the occurrence of four enzymes with amylolytic activity in the carnivorous spiny lobster *P*. *argus* [11]. However, two of these forms were extremely occasional and most individuals of *P*. *argus* had one or two isoforms. There is a wide disparity in the number of α-amylase isoforms in crustaceans, with five or six isoforms in some species [18], and up to ten in some shrimps if pooled glands are used [52], while only one or two isoforms in individual spiny lobsters ([11], this work) and other crustaceans [18]. Few α-amylase isoforms in *P*. *argus* agree with the idea that omnivorous crustaceans have higher number of isoforms than carnivorous species [18]. However, while it is generally accepted that the presence of digestive isoenzymes enables organisms to digest a broad range of substrates in a broad range of environmental conditions, the physiological meaning of the isoenzyme richness in crustaceans has been not evaluated. In very distant groups such as insects, mollusks and birds, α-amylase polymorphism plays an important role in the variation of biological traits such as feed conversion and growth rate due to differences in isoform activity [13,23,24]. This work demonstrated *in vitro* that variation in carbohydrate digestion in a decapod crustacean partially relies on α-amylase isoenzyme composition. Interestingly, the most frequent isoenzyme (and resultant phenotype) is the one of less digestion efficiency. It should be noted that one assumption of our approach is that phenotypic or activity differences in other activities involved (i.e., other starch degrading enzymes) among individual are similar or equally distributed among the experimental groups. Nutritional studies in different spiny lobsters have provided evidence that a gradual digestion of carbohydrates and liberation of glucose to the hemolymph positively impacts their post-absorptive utilization [21, 22]. Thus, amylase polymorphism in lobster may be influenced by selective forces toward less carbohydrate digestion. The effects of these differences still need further *in vivo* examination.

We found only one genomic sequence for α-amylase in lobster. Given that at least three different genes have been reported in an ancient species, the shrimp *Litopenaeus vannamei* [51], our finding indicates that gene loss has occurred through evolution of α-amylase in crustaceans. It is accepted that propensity of a gene to be lost during evolution is related with its biological importance [53] but there is no genomic information in other crustaceans to determine when and how often this has occurred, or if there is any relationship with their feeding habits. In humans, salivary α-amylase gene copy number is higher in high-starch populations than in low-starch populations, evidencing the correlated evolution of diet and α-amylase genes in higher vertebrates [16]. The increase in pancreatic α-amylase gene copy number in dogs respect to wolves also evidences the correlated evolution of α-amylase genes and diet (from carnivorous to starch rich diet through domestication) [15]. In arthropods such as insects, there is variation in the number of α-amylase genes even within the same genera (e.g., two to seven in *Drosophila*, [50] but as in crustaceans, the relation with diet is not totally understood. An interesting finding of this study was the absence of intronic sequences in the lobster gene, which revealed that genome simplification for α-amylase in lobster included extreme intron loss. This is not unusual, as loss of the ancestral intron in one of the α-amylase genes of *Drosophila* has occurred independently in several *Drosophila* linages leading to intron-less genes [50,54,55]. Actually, insect *Amy* genes have undergone mostly intron losses [56]. The loss of introns may be not related with the feeding habits but result from lack of constrains for variation in these regions due to no regulatory or alternative splicing roles in the α-amylase gene [56].

In correspondence with the single α-amylase gene sequence we identified in lobster, we could only describe a single transcript for the enzyme. Exactly the same single transcript was regularly cloned from several individual having one or the two isoenzymes as previously identified by starch zymograms. The transcript we identified has high sequence identity with α-amylases from other decapods such as *L*. *vannamei* (79%) and *M*. *japonicus* (78%), but also high (> 60%) with α-amylases from phylogenetically distant groups such as humans. Accordingly, the corresponding enzyme (513 aa) has a high amino acid conservation respect to other α-amylases, probably related with functional contains on the 3D structure and functional sites. It is known that the α-amylase family has seven conserved regions [57,58,59,60]. The comparison of amino acid sequence of lobster enzyme and other α-amylases showed a high similarity in conserved regions I to VI, but region VII was not identified. The seventh region is known to be less conserved among the family and thus difficult to identify [57]. Ten cysteines residues were observed in PaAmy as occur in α-amylases from other arthropods [61,62]. Eight of these cysteines are conserved in vertebrate α-amylases [63]. The additional two residues in crustaceans and other invertebrates (C436 and C459, PaAmy numbering) enable a fifth disulfide bridge, and maybe related with differences in activity during temperature adaptation [62]. The predicted isoelectric point (pI) 4.93 for the lobster enzyme is similar to that found for shrimp α-amylase [62], but one unit lower than pI of α-amylases from fish [64].

Thus, despite zymograms of lobster α-amylases suggest the presence of codominant alleles at a single locus, we could not explain this at the gene or transcript level. In the porcine pancreas a similar situation was found, were all clones analyzed possessed the same nucleotide sequence suggesting the existence of a single transcribed gene coding for α-amylase, despite two isoenzymes are known to occur [59]. Among decapod crustaceans, the structure of α-amylase genes is known only for shrimp and either does not provide full explanation for the observed number of active forms of the enzyme. Three α-amylase genes were found in the shrimp *L*. *vannamei* [51] but eight α-amylase isoforms can be observed by electrophoresis [62].

Thus, we examined the possibility that limited proteolysis of the single enzyme by digestive proteases would occur giving rise the two active isoforms found in some individuals or the fast migrating form in most individuals. The rational for this hypothesis is that we predicted several putative cleavage sites for digestive proteases, mainly trypsin, in the surface of the lobster α-amylase. However, the incubation of lobster extracts containing the isoform of slower electrophoretic mobility with all endogenous proteases did not yield a second active form nor modified the electrophoretic mobility of the enzyme. Neither could we demonstrate that the bovine trypsin is able to cleave the isolated lobster enzyme. Our observations pointed that the lobster α-amylase is highly resistant to proteolysis probably due to the neighboring of the predicted cleavage sites. In two tilapia species, *Oreochromis niloticus* and *Sarotherodon melanotheron*, even when it was demonstrated that the bigger α-amylase isoenzyme is converted through time into the smaller, proteolysis could not explain this behavior as adding a protease-inhibitor cocktail to fresh tissue did not affect the isoform pattern [65]. Alternatively, although it has been suggested before that glycosylation cannot explain the high degree of α-amylase polymorphism in some crustaceans [18], it may explain the few isoenzymes in lobster. Glycosylation is thought to be the cause of several forms of the human salivary α-amylase [66]. We predicted one site for *N*-glycosylation (N429) in the lobster enzyme. Interestingly, major *N*-glycosylation sites in human salivary and pancreatic α-amylases are N427 and N476 [67]. Thus, differences in glycosylation at this single site (N429), seem a plausible source of α-amylase polymorphism in lobster. This hypothesis is supported by the fact that the lobster isoforms have a clear instead of smeared appearance after electrophoresis. We demonstrated that the two isoenzymes in lobster arise by differences in glycosylation and, by using gastric juice samples, that this is a naturally occurring process instead an artifact of digestive gland homogenization.

Although many studies have characterized α-amylases from crude extracts of different organisms, just a few have purified the enzyme prior to characterization in non-insect animals. In this work, one form of α-amylase from *P*. *argus* was purified to homogeneity by a combination of size exclusion and anion exchange chromatography, as judged by SDS-PAGE, native-PAGE and starch zymography. The apparent molecular weight of the purified lobster α-amylase (60 kDa), determined by SDS-PAGE under reducing conditions, is consistent with our estimation from PaAmy sequence (55.5 kDa) taking into account it is the glycosylated form of the enzyme.

It is well known that ions such as sodium, chloride and calcium, are important for the activity and stability of α-amylases. Lobster α-amylase activity increased with NaCl up to the optimum, and then only slightly declined at higher concentrations as observed for other α-amylases [19,68,69]. Optimum NaCl concentration for the lobster enzyme was 0.3 mM and activity remained high even at salt concentrations higher than those found in sea water (~0.5–0.6 M). We also observed an increment in α-amylase activity with increased concentrations of CaCl_2_ up to 25 mM, while the activity was not affected with subsequent increases. Similar results were found in α-amylases from other invertebrates [70,71], although in some insects high CaCl_2_ concentration inhibited α-amylase activity [72]. In the case of lobster and other carnivorous crustaceans, calcium concentrations higher than in sea water (~10 mM) are expected in the digestive tract due to the ingestion of other crustaceans and mollusks with calcareous exoskeleton, and this appears to increase α-amylase activity in lobster up to 25 mM CaCl_2_.

Our findings on the optimal pH for the lobster α-amylase (pH 5.5) agree with values obtained before from crude extracts [11] and other invertebrate purified α-amylases [19,69], with the pH of gastric juice in different spiny lobsters (e.g., 6.4 to 5.8 [8,11,73]), and with a pH allowing of high enzyme stability. The strong reduction of α-amylase activity we observed below pH 4 was also found in *P*. *argus* crude extracts [11] and in α-amylases from distant groups (e.g., human salivary α-amylase [74]), in agreement with high sequence and structure conservation. Also, the relationship between stability of the lobster α-amylase and temperature is consistent with those observed for other α-amylases [69]. In addition, using CNP-G3 as the substrate we demonstrated that the lobster α-amylase has lower *K*m (0.36 mM) than pancreatic and salivary human α-amylases (1.15 mM) [75]. This indicates that the lobster enzyme saturates at low substrate concentrations and may be a way to control activity in the presence of high carbohydrate loads. However, *K*m values reported for a few of other crustaceans were calculated using starch as the substrate and thus preclude us to make valid comparisons.

On the other hand, mechanisms for regulating digestive enzymes are largely unknown in invertebrates [76]. Research in mollusks [77,78] and insects [79,80] pointed that external factors such as diet have significant effects on the regulation of α-amylase, mostly at the transcription level. We demonstrated that although gene and isoenzyme patterns in the lobster are simpler than in omnivorous crustaceans, α-amylase activity is also tightly regulated by the carbohydrate content of diet. We drew this conclusion from our observations on the similar gene expression and activity in lobsters feeding on different starches at 30% inclusion level, but an increase both in expression and activity when fed on fish muscle (~2 to 5% glycogen). Thus, although it is currently believed that carnivorous species have low enzyme flexibility, we showed that transcriptional and activity flexibility was retained by the lobster enzyme. Our findings agree with previous studies in other lobsters showing adaptation of α-amylase activity to dietary carbohydrates [81].

The release of free glucose into the gastric juice of lobsters occurs soon after ingestion [22], and may play a role in α-amylase regulation. While in some insects free glucose suppress α-amylase activity, no glucose repression (or starch induction) has been observed in others [82], indicating that this is a species-specific phenomenon. An interesting finding in this study is that even when significant differences were found in expression and activity of α-amylase in the digestive gland, all lobster exhibited similarly high level of α-amylase activity in the gastric juice prior to feeding. Given that α-amylase expression is transcriptionally regulated by the carbohydrate content of diet, high α-amylase activity in the gastric juice of fasted lobsters may work as an early sensor of dietary carbohydrates: high content of dietary carbohydrates rapidly produces high amount of free saccharides and this in turns ultimately down-regulates α-amylase gene expression. This hypothesis needs further experimental validation. In the Pacific oyster *Crassostrea gigas* the adaptation to high carbohydrate content in diet is achieved by a different mechanism, such as the up-regulation of an isoform with higher *K*m [78]. Extreme cases have been reported in some crustaceans, where disappearance of some isoforms occurs in the presence of high dietary starch or glycogen [68].

The high conservation of lobster α-amylase respect to other animal α-amylases indicates there are several structural and functional constrains for extensive enzyme variation. Taking together this consideration with our present results, we concluded that gene/transcript/isoenzyme simplification, post-translational modifications and low *K*m, coupled with a tight regulation of gene expression, have arose during evolution of α-amylase in the carnivorous lobster to control excessive carbohydrate digestion in the presence of an active α-amylase.

## Supporting Information

S1 Fig Phylogenetic relationships among α-amylases from different taxa, including the lobster *Panulirus argus*. The tree was constructed with the NJ method. Only bootstrap values higher than 50% are shown on each branch. Species and accession numbers are shown in the tree.
(TIF)

S2 Fig Alpha-amylase expression in the digestive gland and other tissues of the lobster *Panulirus argus*. Gene expression was calculated relative to EF1α. qPCR products were also analyzed by electrophoresis on 2% agarose gel stained with GelRed. NC: negative control without template.
(TIF)

S1 Table Primers used in this study for cDNA cloning, qPCR, and DNA PCR of *Panulirus argus* α-amylase. (DOC)

S2 Table Formulation (%) and proximate composition of the experimental diets. (DOC)
