# Supplementary figures and images for: Molecular, Biochemical, and Dietary Regulation Features of α-Amylase in a Carnivorous Crustacean, the Spiny Lobster Panulirus argus
Source: PLoS One. 2016 Jul 8;11(7):e0158919. doi: 10.1371/journal.pone.0158919 (PMC4938498; doi:10.1371/journal.pone.0158919)

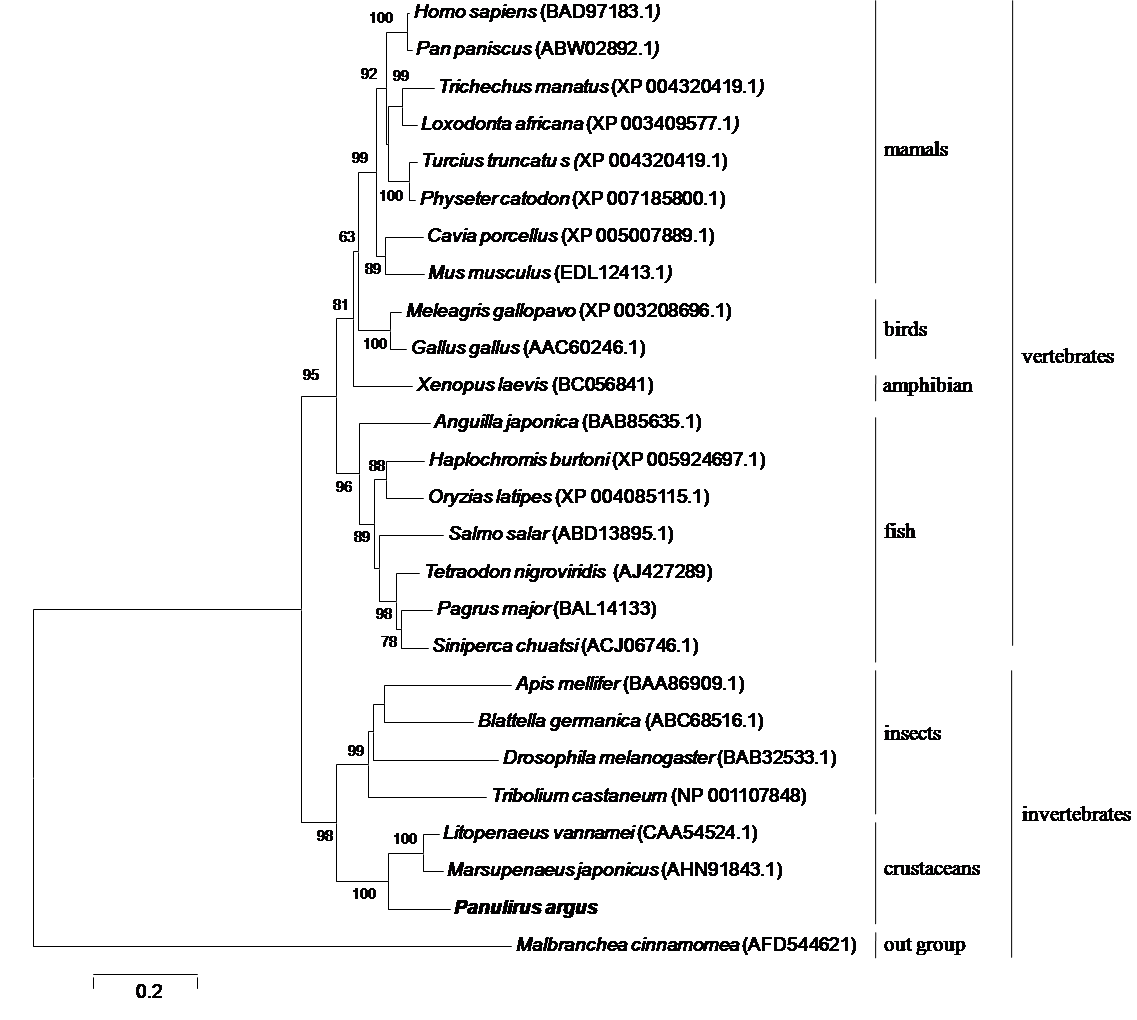

Supplement: S1 Fig — The tree was constructed with the NJ method. Only bootstrap values higher than 50% are shown on each branch. Species and accession numbers are shown in the tree. (TIF) [file pone.0158919.s001.tif]

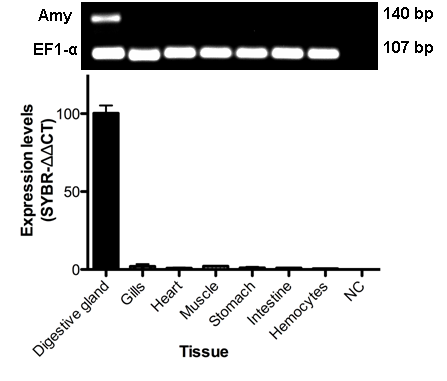

Supplement: S2 Fig — Gene expression was calculated relative to EF1α. qPCR products were also analyzed by electrophoresis on 2% agarose gel stained with GelRed. NC: negative control without template. (TIF) [file pone.0158919.s002.tif]
