# Supplementary material for: Molecular, Biochemical, and Dietary Regulation Features of α-Amylase in a Carnivorous Crustacean, the Spiny Lobster Panulirus argus
Source: PLoS One. 2016 Jul 8;11(7):e0158919. doi: 10.1371/journal.pone.0158919 (PMC4938498; doi:10.1371/journal.pone.0158919)
Supplement: S1 Table — (DOC) [file pone.0158919.s003.doc]

**S1 Table.** **Primers used in this study for cDNA cloning, qPCR, and DNA PCR of *Panulirus argus* α-amylase.**

| **Primer name** | **Nucleotide sequence** | **Size amplified** |
| --- | --- | --- |
| **Degenerate primers** |  |  |
| Pa AmyFw1 | 5’- ATBGTSCAYYTBTTYGARTGG -3’ | 1250 bp |
| PaAmy Rv1 | 5’- TVACVNBCTTBCCMGTGCA-3’ |
| Pa AmyFw2 | 5’- GGCCDTGGTGGGARMGDTAC -3’ | 1050 bp |
| PaAmy Rv2 | 5’- CCRGARATSACRTCRCAGTA -3’ |
| PaAmy Fw3 | 5’- GCYDSCAARCACATGTGGCC -3’ | 550 bp |
| PaAmy Rv3 | 5’- TTNCGGAADNBVACCATGTT -3’ |
| **5′ RACE** |  | **Position in cDNA** |
| Pa- 5’outer | 5′- CTCCAAGATCGATGACCTCTTG -3′ | 780 - 801 |
| Pa- 5’inner 1 | 5′- GCTGGTCAAGGTGTTGAGGTAG -3′ | 629 - 650 |
| Pa- 5’inner 2 | 5′- TGAAGTCGAAGGCAGAGTAAGG -3′ | 474 - 495 |
| **3′ RACE** |  |  |
| Pa- 3’outer | 5′- CGATCCTCCAAGTGGTACAAGA -3′ | 1016 - 1038 |
| Pa- 3’inner 1 | 5′- CGGGTCATGTCCTCCTTCTACT -3′ | 1080 - 1101 |
| Pa- 3’inner 2 | 5′- GCAGATCTACAACATGGTCCAA -3′ | 1241 - 1262 |
| **qPCRprimers** |  |  |
| EF1-α Fw | 5’-CCAGTAGACAAACCACTTCG-3’ | 532 - 551 |
| EF1-α Rv | 5’-CATACCTGGCTTCAAGATGC-3’ | 620 - 639 |
| Pa-qPCR-FwAMY | 5’- GAGTGACGGAGTTCAAGTACGG -3’ | 841 - 862 |
| Pa-qPCR-RvAMY | 5’- GTCGTGGTTGTCGATGAAGAC -3’ | 980 - 1000 |
| **gDNAcloning** |  |  |
| Pa-gDNA Fw1 | 5’-TAGATGGTGGGCAGGAAGG -3’ | 5 - 23 |
| Pa-gDNA Fw2 | 5’-GTACCAGGATGAGAAGTCCCG -3’ | 236 - 256 |
| Pa-gDNA Fw3 | 5’-CCTTACTCTGCCTTCGACTTCA -3’ | 474 - 495 |
| Pa-gDNA Fw4 | 5’-AGGCAAGATCAGGGACTACCTC -3’ | 614 - 635 |
| Pa-gDNA Fw5 | 5’-CCATCACTTCGTCCCAGTACG -3’ | 808 - 828 |
| Pa-gDNA Fw6 | 5’-CATTCCGATCCTCCAAGTGG -3’ | 1012 - 1031 |
| Pa-gDNA Fw7 | 5’-CTTCTGCAGGGGCGACAG -3’ | 1328 - 1345 |
| Pa-gDNA Rv7 | 5’-TCTTGTAGGAGACGGGCTGG -3’ | 275 - 294 |
| Pa-gDNA Rv6 | 5’-TGAAGTCGAAGGCAGAGTAAGG -3’ | 474 - 495 |
| Pa-gDNA Rv5 | 5’-ACTTGCCGTACTTGAACTCCG -3’ | 847 - 867 |
| Pa-gDNA Rv4 | 5’-TCTTGTCGTGGCCGTTCTC -3’ | 1116 - 1134 |
| Pa-gDNA Rv3 | 5’-GTCCCACCAGTCCTTCATGT -3’ | 1288 - 1307 |
| Pa-gDNA Rv2 | 5’-GGTCGTTGTTGATGGCGATG -3’ | 1352 - 1371 |
| Pa-gDNA Rv1 | 5’-TTACAAGGGACTTCAGGCTCAG -3’ | 1702 - 1723 |
