# Supplementary material for: Molecular, Biochemical, and Dietary Regulation Features of α-Amylase in a Carnivorous Crustacean, the Spiny Lobster Panulirus argus
Source: PLoS One. 2016 Jul 8;11(7):e0158919. doi: 10.1371/journal.pone.0158919 (PMC4938498; doi:10.1371/journal.pone.0158919)
Supplement: S2 Table — (DOC) [file pone.0158919.s004.doc]

**S2 Table.** **Formulation (%) and proximate composition of the experimental diet**s.

| **Ingredients** | **Rice diet** | **Wheat diet** | **Maize diet** |
| --- | --- | --- | --- |
| Fish meal^a^ | 35 | 31 | 35 |
| Squid meal^a^ | 17 | 13.2 | 17 |
| Gelatin^b^ | 5 | 5 | 5 |
| Wheat flour^c^ | - | 40.9 | - |
| Maize starch^d^ | - | - | 30 |
| Rice starch^e^ | 30 | - | - |
| Fish oil^f^ | 1.9 | 1.9 | 1.9 |
| Lecithin^g^ | 2 | 2 | 2 |
| Cholesterol^h^ | 1 | 1 | 1 |
| Vit & Min premix^i^ | 1 | 1 | 1 |
|  |  |  |  |
| Phosphate/carbonate^j^ | 2 | 2 | 2 |
| Attractants^k^ | 1 | 1 | 1 |
| Talc^c^ | 4.10 | 1 | 4.1 |
| Total | 100 | 100 | 100 |
| Crude protein | 44.9 | 43.6 | 44.9 |
| Crude lipid | 4.9 | 5.4 | 4.9 |

Pellets contain 10-12% of water

^a^ Jack mackerel meal: 79.1% proteins, 16.8% lipids, 5.5% moisture; Squid meal: 76.6% proteins, 10.8% lipids, 8.7% moisture

^b^Sigma-Aldrich (G2500)

^c^Commercially available regular feedstuff

^d^Indias, G.R. Baldinelli, Argentina

^e^BDH (30263), Merck Chemicals Ltd.

^f^Fisheries Research Center Laboratory, Havana, Cuba

^g^Calbiochem (429415), Merck Chemicals Ltd.

^h^Sigma-Aldrich (C8667)

^i^Premix from DIBAQ-Aquaculture, Segovia, Spain, containing (per kg of feed): vitamin A 15,000 IU, vitamin D3 3000 IU, vitamin E 180 mg, vitamin K 15 mg, vitamin B1 37.5 mg, vitamin B2 37.5 mg, vitamin B6 24.75 mg, vitamin B12 0.045 mg, vitamin H 1.14 mg, D-pantothenic acid 120 mg, nicotinic acid 225 mg, vitamin C 300 mg, folic acid 11.24 mg, Inositol 112.5 mg, zinc 75 mg, selenium 0.3 mg, magnesium 86.25 mg, copper 2.25 mg, manganese 22.5 mg, iodine 7.5 mg, iron 3 mg, cobalt 0.3 mg

^j^Dicalcium phosphate/Calcium carbonate (1:2), Santa Cruz Fish Feed Factory, Camagüey, Cuba

^k^Taurine (Sigma-Aldrich, T0625) 500 mg/Kg diet, Glycine (Sigma-Aldrich, G8898) 500 mg/Kg diet
